# Proteome remodelling by the stress sigma factor RpoS/σ^S^ in *Salmonella*: identification of small proteins and evidence for post-transcriptional regulation

**DOI:** 10.1038/s41598-017-02362-3

**Published:** 2017-05-18

**Authors:** Magali Lago, Véronique Monteil, Thibaut Douche, Julien Guglielmini, Alexis Criscuolo, Corinne Maufrais, Mariette Matondo, Françoise Norel

**Affiliations:** 1 Institut Pasteur, Laboratoire Systèmes Macromoléculaires et Signalisation, Département de Microbiologie, rue du Dr. Roux, 75015 Paris, France; 2 CNRS ERL6002, rue du Docteur Roux, 75015 Paris, France; 3 Université Paris Diderot, Sorbonne Paris Cité, Cellule Pasteur, Paris, rue du Dr. Roux, 75015 Paris, France; 4 Institut Pasteur, Unité de Biochimie des Interactions Macromoléculaires, Département de Biologie structurale et Chimie, rue du Dr. Roux, 75015 Paris, France; 5 Institut Pasteur, Unité de Spectrométrie de Masse Structurale et Protéomique, Département de Biologie Structurale et Chimie, UMR3528, rue du Dr. Roux, 75015 Paris, France; 6 Institut Pasteur, Bioinformatics and Biostatistics Hub, C3BI, USR 3756 IP CNRS, rue du Dr. Roux, 75015 Paris, France

## Abstract

The RpoS/σ^S^ sigma subunit of RNA polymerase is the master regulator of the general stress response in many Gram-negative bacteria. Extensive studies have been conducted on σ^S^-regulated gene expression at the transcriptional level. In contrast, very limited information regarding the impact of σ^S^ on global protein production is available. In this study, we used a mass spectrometry-based proteomics approach to explore the wide σ^S^-dependent proteome of the human pathogen *Salmonella enterica* serovar Typhimurium. Our present goals were twofold: (1) to survey the protein changes associated with the Δ*rpoS* mutation and (2) to assess the coding capacity of σ^S^-dependent small RNAs. Our proteomics data, and complementary assays, unravelled the large impact of σ^S^ on the *Salmonella* proteome, and validated expression and σ^S^ regulation of twenty uncharacterized small proteins of 27 to 96 amino acids. Furthermore, a large number of genes regulated at the protein level only were identified, suggesting that post-transcriptional regulation is an important component of the σ^S^ response. Novel aspects of σ^S^ in the control of important catabolic pathways such as myo-inositol, L-fucose, propanediol, and ethanolamine were illuminated by this work, providing new insights into the physiological remodelling involved in bacterial adaptation to a non-actively growing state.

## Introduction

*Salmonella enterica* serovar Typhimurium (hereafter referred as *Salmonella* or *S*. Typhimurium) is a facultative intracellular bacterial pathogen capable of colonizing a wide range of hosts, including humans and many animal species. This serotype is ubiquitous in the environment, and its capacity to adapt to many environmental conditions depends on its ability to integrate various environmental signals to coordinate gene expression appropriately. The alternative sigma factor σ^S^ (also called σ^38^ or RpoS) of RNA polymerase (RNAP) controls a global adaptive response allowing many Gram-negative bacteria to survive nutrient deprivation and environmental stresses^1–3^. σ^S^ also contributes to virulence and biofilm formation of *S*. Typhimurium^3–5^. In contrast to the housekeeping sigma factor σ^70^, σ^S^ is almost undetectable in early exponential phase. Its expression is induced in stationary phase, or in response to various stresses, by a fine-tuned combination of transcriptional, translational and proteolytic controls^2, 3^. σ^S^ and σ^70^ bind to almost identical –35 and –10 promoter elements, but σ^S^ is more tolerant than σ^70^ with respect to deviations from the consensus promoter sequences, especially in the −35 region^3, 6–10^. The activities of the σ^S^- and σ^70^- dependent RNAP can be modulated by additional DNA-binding regulatory proteins, which can also contribute to σ factor selectivity at a given promoter^2, 3^.

Regulatory action of σ^S^ has been extensively studied at the transcriptional level. Global transcriptomic analyses have revealed that σ^S^ controls, directly or indirectly, the expression of 10–20% of the genome of *Escherichia coli* K12^3, 7, 11–15^. We have recently used RNA-sequencing to unravel the σ^S^-dependent transcriptome in *S*. Typhimurium ATCC14028^16^. These studies have revealed a major effect of σ^S^ on the remodelling of metabolism and membrane functions, and have highlighted the importance of down-regulation of gene expression by σ^S^ ^16, 17^. Negative regulation by σ^S^ is an active process requiring σ^S^ binding to DNA^17^. Mechanisms of repression could be direct in some cases^14, 17, 18^, through competition with σ^70^ for promoter binding^14, 17^, or indirect, *via* transcription activation of repressor molecules^3, 16, 17^. In particular, we have unravelled σ^S^-regulation of a large number of small RNAs (sRNAs)^16^, among which some might endow σ^S^ with repressor functions, by affecting mRNA stability and/or translation or protein stability.

Compared to transcriptomic data, very limited information regarding the effect of σ^S^ on global protein production is available. The proteome of wild type and Δ*rpoS* strains have been compared in a few studies by using bi-dimensional gel electrophoresis^3, 19–22^. Although the expression of dozens of proteins correlated with transcriptomic data, only a small fraction of the proteome was assessed in those studies, and the σ^S^-dependent proteome was largely underestimated. Comparative proteomic profiling using mass spectrometry (MS) is a powerful tool for investigating differences in global protein abundance that occur in response to a mutation, or a specific condition. In bacteria, MS-based proteomics is under-represented when compared to transcriptomic studies and has not been used, to our knowledge, to characterize the σ^S^-dependent proteome.

In this study, we performed label-free MS-based relative quantification of protein abundance in the wild type and Δ*rpoS* strains of *S*. Typhimurium. Our present goals were twofold: (1) to survey the protein changes associated with the Δ*rpoS* mutation and (2) to assess the coding capacity of σ^S^-dependent small RNAs (sRNAs) revealed by our transcriptomic analyses^16^ (Supplementary Fig. S1). Our proteomics data, and complementary assays, unravelled the large impact of σ^S^ on the *Salmonella* proteome in stationary phase, and validated translation and σ^S^ regulation of more than twenty sRNAs. As expected, the majority of genes up-regulated by σ^S^ at the protein level were also up-regulated at the transcript level^16^. In sharp contrast, our data revealed a large number of genes down-regulated at the protein level, but not at the transcript level, suggesting that post-transcriptional regulation plays a larger role in σ^S^ gene regulation than previously recognized. In addition, this study unravelled new facets of σ^S^ in metabolism rewiring during bacterial adaptation to a non-actively growing state. The complexity of the σ^S^ regulatory network and its impact on cell physiology, revealed by this study, points to a key role of σ^S^, at the transcriptional and post-transcriptional levels, in maintaining the delicate balance between cellular resistance during quiescence and re-growth potential, under diverse environmental conditions.

## Results and Discussion

### STnc1330, STnc1110 and IsrI are coding sRNAs

**Figure 1:**
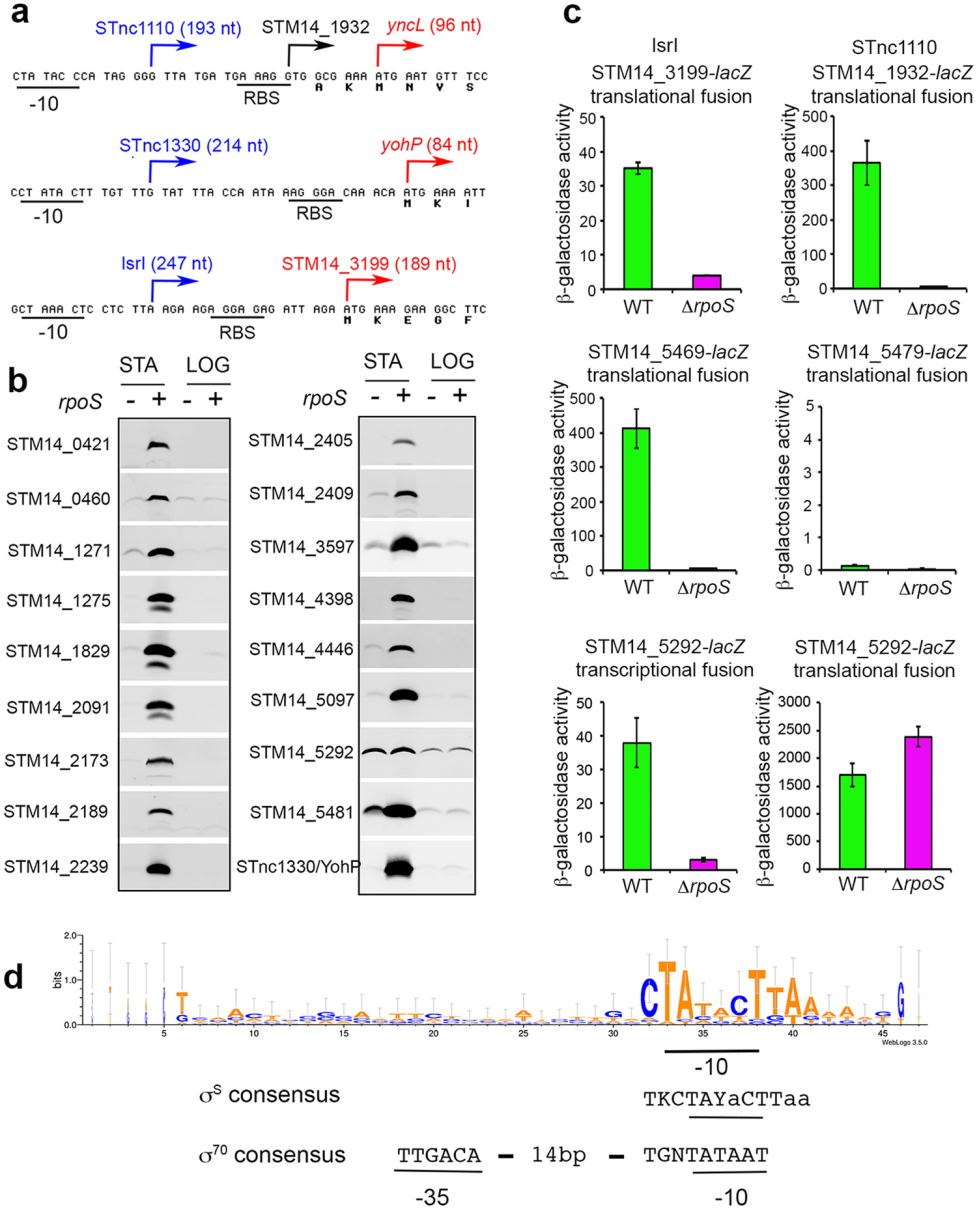
Expression and σ^S^ regulation of proteins encoded by sRNAs. (**a**) DNA sequences corresponding to the 5′ end and upstream regions of the STnc1110, STnc1330, and IsrI sRNAs genes are shown. The −10 promoter regions and potential ribosome binding sites (RBS) are underlined. STnc1110 and STnc1330 sRNAs are predicted to encode protein homologous to the YncL and YohP proteins of *E*. *coli*, respectively (see Supplementary Fig. S2). Broken arrows indicate the 5′ ends of sRNAs (in blue) and ORFs (in red). The translation start of STM14_1932/*yncL* is probably located three codons downstream of the annotated GTG start codon. STM14_3199 is conserved in several bacterial species but not in *E*. *coli* K12 (Supplementary Fig. S3). (**b**) Production and σ^S^-regulation of proteins encoded by sRNAs was assessed by immunodetection of 3xFlag-tagged proteins in exponential (LOG, OD_600_ of 0.3) and stationary phase (STA, 18 h, OD_600_ of 4) LB cultures of wild type and *rpoS Salmonella* strains (VF6910 and VFC331). The amount of proteins in whole-cell lysates was determined and equal amounts of total proteins were loaded in each slot of the gel slices (between 5 and 20 μg, depending on the protein studied). Reversible Ponceau staining of the membrane was used to check proteins transfer. Similar results were obtained in at least two independent experiments. (**c**) Expression of transcriptional and translational *lacZ* fusions in predicted small ORFs. Expression of the indicated *lacZ* fusions was followed in *Salmonella* wild type and Δ*rpoS* strains grown 18 h in LB. Bar graphs represent the mean β-galactosidase activity, and error bars represent standard deviation of at least three independent experiments. (**d**) Promoter features of the σ^S^-dependent coding sRNAs. Sequence logo was generated with promoter sequences listed in Supplementary Fig. S7 and the WebLogo application (http://weblogo.threeplusone.com/create.cgi). Consensus sequences for promoters preferentially recognized and transcribed by σ^S^ and σ^70^ ^3, 8, 10^ are shown below the logo. The −35 element is less conserved in σ^S^-dependent promoters and is variable in its sequence and location^3, 8, 10^. The most conserved nucleotides are indicated in capital letters. Y denotes a pyrimidine (T/C). K stands for T/G.

Our recent RNA-sequencing data in *S*. Typhimurium ATCC14028^16^ revealed a number of transcripts showing high relative abundance and strong σ^S^-dependency in stationary phase, including the σ^S^-dependent sRNAs STnc1110, SdsR, STnc1330, IsrI, and SraL^16^ (Supplementary Fig. S1). Regulatory functions have been described for SdsR^23, 24^ and SraL^25^, but not yet for IsrI, STnc1110 and STnc1330. Surprisingly, open reading frames (ORFs) showing good potential ribosome binding sites were predicted in the sequences of IsrI^26^, and of STnc1110 and STnc1330 which originally were considered non-coding^27^ (Fig. 1a and Supplementary Fig. S2). Moreover, whereas these sRNAs have been reported to be specific to *Salmonella* ^28, 29^, the predicted ORFs in STnc1110 and STnc1330 were actually orthologs of the *E*. *coli yncL* and *yohP* genes, respectively (Supplementary Fig. S2). In *E*. *coli*, *yncL* and *yohP* direct the synthesis of hydrophobic membrane proteins of 31 and 27 amino acids, respectively, and of unknown function^30, 31^. The predicted ORF in *isrI*, STM14_3199, encodes a product of 62 amino acids (Supplementary Fig. S3). IsrI^26^ is located on the Gifsy-1 prophage in *S*. Typhimurium ATCC14028, SL1344 and LT2^27^. Interestingly, STM14_3199 is paralogous to STM14_1447 (75% identity at the amino acid sequence level, Supplementary Fig. S3). STM14_1447 is carried on the Gifsy-3 prophage, which is present in ATCC14028, but absent from strains LT2 and SL1344^32^. Homologs of STM14_3199 were found in a few other enterobacterial species (Supplementary Fig. S3).

To assess the coding capacity of IsrI, STnc1110 and STnc1330, a DNA sequence encoding a 3xFlag epitope was inserted at the 3′ end of each of the predicted ORFs, and production of the flag-tagged proteins was followed by immunodetection, using an anti-flag antibody. A 3xFlag-tagged YohP protein was produced in high amounts in ATCC14028, and was almost undetectable in the Δ*rpoS* mutant (Fig. 1b). Production of YohP was also strongly dependent on the growth phase (Fig. 1b). Although no flag-tagged protein was detected for STM14_1932/*yncL* and STM14_3199, translational STM14_3199-*lacZ* and STM14_1932-*lacZ* fusions were expressed in ATCC14028, and their expression required σ^S^ (Fig. 1c). The STM14_3199 and STM14_1932 flag-tagged products may be unstable, or produced at levels too low to be detected under the experimental conditions used. Altogether, these data indicated that STnc1330, and likely STnc1110 and IsrI, are coding sRNAs.

### Additional coding sRNAs tightly controlled by σ^S^

Besides STnc1110, STnc1330 and IsrI, twenty-nine additional small transcripts, abundant in stationary phase and tightly controlled by σ^S^, were assigned to small annotated ORFs (Supplementary Figs S1 and S4, Supplementary Table S1). To validate these data, we first examined the genomic context and sequence features of these ORFs. This analysis suggested that transcripts assigned to the small ORFs STM14_0419, STM14_1559, and STM14_5096, and the pseudogene STM14_1274, more likely correspond to long 5′ UnTranslated Regions (UTR) of the σ^S^-dependent genes STM14_0421, STM14_1558, STM14_5097, and STM14_1275, respectively (Supplementary Fig. S4 and Table S1). This hypothesis is consistent with the non-canonical start codons and lack of ribosome binding sites for the putative ORFs STM14_0419, STM14_1559 and STM14_5096, and the location of transcription start sites and RNA reads in these regions (Supplementary Fig. S4). Of the remaining twenty-five small annotated ORFs, only six have been functionally characterized, at least to some extent (*ecnB*, *yqaE*, *chaB*, *osmB*, *yahO* and *yciG*, Supplementary Table S1 and references therein). Nineteen ORFs were putative or of unknown function (Table 1). Examination of their sequence features, and alignment of their predicted amino acid sequences with that of identified homologs in other bacterial genomes, prompted us to re-annotate the start codon of STM14_2239, STM14_2409, and STM14_5481 (Table 1, Supplementary Fig. S5, Supplementary Dataset S1). The uncharacterized ORFs *ymdF* and STM14_1829 are paralogous to *yciG* (Table 1, Supplementary Fig. S6). YciG^33, 34^ belongs to the group of “hydrophilins”, proteins defined by high glycine content and hydrophilicity index^35^. Interestingly, while some of the uncharacterized small ORFs appear to be restricted to bacteria of the *Enterobacteriaceae* family, others are more widely distributed (Table 1 and Supplementary Dataset S1) and might be involved in conserved biological processes. In the following part of the work, a LC-MS-based proteomics approach was used to validate production and σ^S^ regulation of these small proteins, and to assess, to our knowledge for the first time, global effects of σ^S^ at the protein level in *Salmonella*.

**Table 1:** Uncharacterized ORFs of less than 300 nucleotides tightly controlled by σ^S^.

Gene in ATCC14028	Protein^a^			*E*. *coli* K12 homolog^b^		Phylogenetic distribution^c^
	Re-annotation and/or features	Size aa	LC-MS id	Name	RpoS^d^	
STM14_0421	—	33	Yes	—		*Enterobacteriaceae*
STM14_0460	—	63	Yes	*yaiA*	12	γ-Proteobacteria
STM14_1271	YccJ-like protein IPR025600	75	Yes	*yccJ*	11–14	γ- Proteobacteria
STM14_2173	Transglycosylase motif IPR007341, 3TM fragments	84		*ymgE*	11–14	Bacteria and Archaea
STM14_2188	—	45	Yes	—		*Enterobacteriaceae*
STM14_2189	—	32		—		*Salmonella*
STM14_2239	Start codon re-annotated, 88% identity with *E*. *coli* YebV, DUF1480 IPR009950	78	Yes	*yebV*	13, 14	*Enterobacteriaceae*
STM14_2405	DUF2525 IPR019669	75	Yes	*yodD*	11–14	*Enterobacteriaceae*
STM14_2409	Start codon re-annotated, 83% identity with *E*. *coli* YodC, DUF2158 IPR019226	61	Yes	*yodC*	11, 13, 14	γ-, α-, β- Proteobacteria
STM14_3597	Sm-like protein IPR010920, pdb 2RA2, SP, Lipo, DUF903 IPR010305	75	Yes	*ygdI*	12–14	Bacteria
STM14_4398	Putative transcriptional regulator, DNA-binding domain IPR010982	96	Yes	*yiaG*	7, 11–14	Bacteria
STM14_4446	79% identity with *E*. *coli* YibT, DNA polymerase III-theta IPR009052	69	Yes	*yibT*	14	*Enterobacteriaceae*
STM14_5097	CsbD like IPR008462, pdb1RYK	70	Yes	*yjbJ*	7, 11–14	Bacteria, Archaea and Eukaryota
STM14_5292	DUF1107 IPR009491	68		*ytfK*	7, 12–14	γ- Proteobacteria
STM14_5469	65% identity with *E*. *coli* YjjZ, 3TM fragments, DUF1435 IPR009885	78		*yjjZ*	13, 14	*Enterobacteriaceae*
STM14_5479	Not translated under the conditions used	44		—		*Salmonella*
STM14_5481	Start codon re-annotated, 98% identity with *E*. *coli* YtjA, 2TM fragments, DUF1328 IPR009760	53		*ytjA*	13, 14	Bacteria and Archaea
**Paralogous genes** ^**e**^						
STM14_1275	KGG repeat IPR019626	55	Yes	*ymdF*	13, 14	Bacteria and Eukaryota
STM14_1829	KGG repeat IPR019626	60	Yes	—		
STM14_2091	KGG repeat IPR019626	60	Yes	*yciG*	12–14	

^a^Information about protein families (DUF), motifs (Interpro IPR), 3D structures (PDB code), predicted α-helical trans-membrane (TM) fragments, signal peptide (SP) and lipoprotein (Lipo) are indicated (Supplementary Methods). The start codon was re-annotated for three ORFs (Supplementary Fig. S5). In most cases, the protein was identified by nLC-MS (Supplementary Table S1 and Datasets S2–S3). ^b^Eight ORFs had annotated homologs in *E*. *coli* K12 and homologs were found for five additional ORFs (Supplementary Fig. S5). ^c^Possible homologs were identified as described in Methods. For each protein, a complete list of homologous sequences is given in Supplementary Dataset S1. ^d^RpoS dependency for expression of *E*. *coli* K12 orthologs in transcriptomic analyses. ^e^STM14_1275 and STM14_1829 are paralogous to STM14_2091 (Supplementary Fig. S6 and Table S1).

### Global effects of σ^S^ on protein abundance in *Salmonella*

A comprehensive quantitative proteomic analysis was performed using the wild type and Δ*rpoS* strains of *Salmonella* ATCC14028, grown in nutrient-rich LB medium to late stationary phase, *i*.*e*. the growth conditions previously used to characterize the σ^S^-transcriptome^16^. Three biological replicates of wild type and Δ*rpoS* strains were grown, and subjected to proteome-wide label-free quantification. After harvesting, cells were lysed, proteins were digested with rLys-C and trypsin, and the resulting peptides were analysed by nLC-MS/MS. A statistical analysis of relative changes in protein abundance between the wild type strain and the Δ*rpoS* mutant was performed using Perseus, the companion software of MaxQuant (see Supplementary Methods for details). A protein was considered as being “present” in a strain, if observed in at least two of the three replicates. Proteins detected in less than two replicates of one strain and in at least two replicates of the other strain, were designated as “exclusive” to that latter strain. Two-sided T-tests of the base-2 logarithm (log_2_) transformed intensity values were employed, using three False Discovery Rates (FDR of 5%, 1% and 0.1%) to identify differentially abundant proteins with different degrees of statistical significance. A complete list of all proteins, along with their relative abundance pattern and sequence coverage is provided in Supplementary Dataset S2. The (log_2_) fold change of the level of each protein in the Δ*rpoS* mutant, with respect to that in wild type strain, is shown. Positive values indicate higher abundance in the mutant than the wild type. Negative values represent lower levels in the mutant than the wild type.

**Figure 2:**
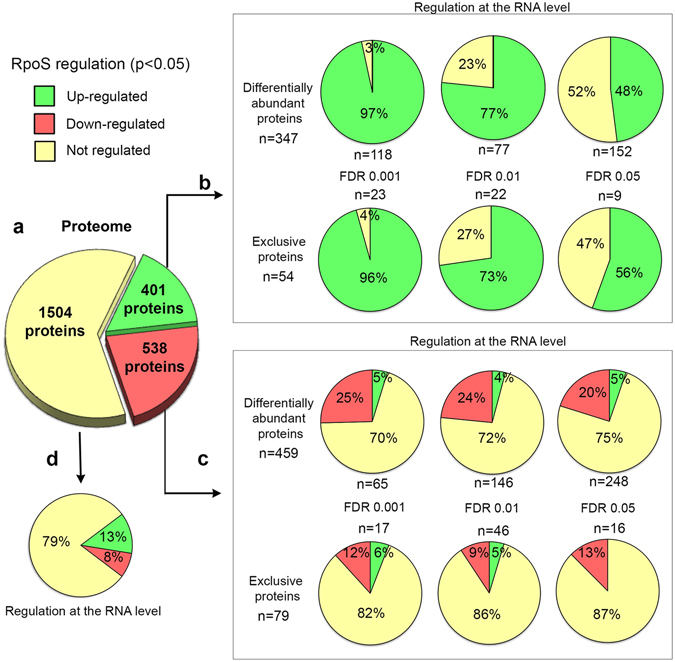
The σ^S^–dependent proteome of *S*. Typhimurium ATCC14028 and comparison with transcriptomic data^16^. *Salmonella* wild type and Δ*rpoS* strains were grown in LB medium to stationary phase in three biological replicates. Total protein was digested and the peptides were analysed by nLC-MS/MS. The pie charts show (**a**) total proteins up- and down-regulated by σ^S^ (p-value < 0.05) and (**b**,**c**,**d**) comparison with transcriptomic data^16^ (see also text and Methods for details). Among the 2444 proteins identified, 939 proteins showed significant differences in abundance between the wild type and Δ*rpoS* strains, including 54 proteins found exclusively in the wild type strain and 79 proteins found exclusively in the Δ*rpoS* mutant (p-value < 0.05, Supplementary Dataset S3). A high correlation was found between the proteome and transcriptome data for proteins up-regulated by σ^S^ (**b**): 97 to 50% (according to the FDR) of the 401 up-regulated proteins were encoded by genes activated by σ^S^ at the RNA level. In sharp contrast, more than 70% of the 538 proteins down-regulated by σ^S^ (**c**) were encoded by genes showing no significant σ^S^ regulation at the transcript levels (p-value > 0.05, Supplementary Dataset S3). For this category of proteins, the degree of correlation between the proteome and transcriptome data sets was not dependent on the FDR. Among proteins showing no σ^S^ regulation, 79% were encoded by genes showing no regulation by σ^S^ at the transcript level (**d**).

A total of 2444 *Salmonella* proteins were identified (Fig. 2a, Supplementary Dataset S2), which corresponds to about 46% coverage of the ATCC14028 proteome. The remaining proteins might not be expressed under the growth condition employed in this study, or they might be expressed at levels too low to be detected. Also, hydrophobic integral membrane proteins are difficult to identify by MS-based proteomics^36^.

Expression of σ^S^ was associated with the significant increase in the level of 401 proteins and a decrease in abundance of 538 proteins (Fig. 2a, Supplementary Datasets S2–S3). For these two groups, the change in the level will be referred to as “up- and down-regulation” regardless of the specific mechanism (or combination of mechanisms) leading to such change. The log_2_ ratios of fold changes were at least 0.68 (representing a minimum fold change value of 1.5). Of note, the log_2_ fold changes in protein abundance were the highest when the FDR was low, and were higher for up-regulated than for down-regulated proteins (Supplementary Dataset S3). The set of 401 up-regulated proteins contained 54 proteins exclusively found in the wild-type strain (Fig. 2b). The set of 538 down-regulated proteins contains 79 proteins found exclusively in the Δ*rpoS* mutant (Fig. 2c).

σ^S^ affected directly or indirectly the abundance of proteins involved in a variety of processes including metabolism, membrane trafficking, regulation, and stress resistance, and of many uncharacterized proteins (Supplementary Dataset S3). These data confirmed at the proteome level the large impact of σ^S^ in bacterial adaptation to a non-growing lifestyle.

### Small uncharacterized proteins in the σ^S^-network

Examination of the proteome data sets allowed identification of protein products for 80% of genes showing strong σ^S^-dependency at the transcript level (Supplementary Table S1). The twelve proteins not identified by LC-MS all contain lysine and arginine residues, and are thus likely sensitive to protease digestion used to generate peptides for LC-MS. However, nine are likely associated with the membrane (Table 1 and Supplementary Table S1) and might not be soluble enough to be detected by this approach.

Interestingly, proteins were identified for more than half of the uncharacterized σ^S^-dependent small ORFs (Table 1 and Supplementary Table S1). Of note, peptides identified by MS allowed discrimination between the *yciG*, *ymdF* and STM14_1829 gene products, despites their high level of sequence conservation (Supplementary Fig. S6). The coding capacity of the uncharacterized small ORFs, for which no protein was identified by LC-MS (STM14_2173, STM14_2189, STM14_5292, STM14_5469, STM14_5479, STM14_5481), was assessed by immunodetection of the corresponding 3xFlag-tagged proteins. Proteins were detected for all of them, except STM14_5469 and STM14_5479 (Fig. 1b). In the case of STM14_5469, canonical translation initiation signals are present (Supplementary Fig. S5), and an STM14_5469-*lacZ* translational fusion was expressed and activated by σ^S^ (Fig. 1c). Thus, in this case, the 3xFlag tag may interfere with the cellular localization and/or the stability of the protein, explaining why it was not detected. No significant expression of the STM14_5479-*lacZ* translational fusion was detected in the growth condition used (Fig. 1c). STM14_5479 does not seem to have a canonical ribosome-binding site (Supplementary Fig. S5), suggesting that the putative ORF might not be translated. We cannot exclude, however, that its translation requires a specific condition. In contrast to STM14_5469, STM14–5479 is conserved in only a few genomes of *Salmonella* (Table 1 and Supplementary Dataset S1).

With the exception of STM14_5292/*ytfK*, a good correlation was found for changes in expression of the uncharacterized σ^S^-dependent small ORFs at the RNA and protein levels (Fig. 1b and Supplementary Table S1). This finding is consistent with transcription activation of these genes by σ^S^. However, many genes transcriptionally activated by σ^S^ can also be transcribed by σ^70^ during exponential growth, either from a unique promoter recognized by the two sigmas, or from different promoters^3, 8^. To assess growth-phase regulation, expression of the 3xFlag-tagged small proteins was examined in logarithmic phase of growth (Fig. 1b). With the exception of YtfK, production of the proteins was strongly dependent on stationary phase, suggesting a tight control by σ^S^ under these conditions. Consistent with this hypothesis, promoter sequences of these genes^28, 37^ showed typical features of canonical σ^S^- promoters in the −10 region^3, 8, 10^, and no conservation of the σ^70^ consensus in the −35 region (Fig. 1d). *E*. *coli* K12 orthologs of the small genes also showed σ^S^ activation in transcriptomic analyses^7, 11–14^ (Table 1), including *ytfK*, which displayed in our studies regulation by σ^S^ at the RNA level only. The impact of σ^S^ on YtfK protein abundance in *E*. *coli* K12 remains to be determined.

### Comparison of the global proteomic and transcriptomic σ^S^ profiles

One of the main issues addressed in this study was to determine to which extent changes induced by the Δ*rpoS* mutation at the transcriptome and proteome levels are correlated. Our proteomics approach identified a protein for about half of genes showing detectable expression by RNA-seq^16^ (Supplementary Dataset S3). The other genes might be poorly transcribed and/or translated, or their products might be unstable or hardly detectable by MS.

For proteins up-regulated by σ^S^, a high correlation was observed for changes in gene expression at the transcript and protein levels, especially for proteins selected with a FDR of 0.1% and 1% (Fig. 2b, Supplementary Dataset S3). Half of the up-regulated proteins selected with a 5% FDR were encoded by genes showing no significant (p-value > 0.05) regulation by σ^S^ at the transcriptional level. This apparent discrepancy could result from some proteins being encoded by genes activated by σ^S^ post-transcriptionally, or genes with a low level of transcription that excluded them from the comparative transcriptomic analysis. It is also possible that some proteins are damaged and degraded in the Δ*rpoS* mutant as a result of increased endogenous stress (*i*.*e*. endogenous oxidative stress may target proteins that are very sensitive to carbonylation and favour their degradation).

Strikingly, a different picture emerged for proteins down-regulated by σ^S^ (Fig. 2c, Supplementary Dataset S3). Most of these proteins were encoded by genes showing no significant regulation by σ^S^ at the RNA level (p > 0.05). Furthermore, about 5% of down-regulated proteins were encoded by genes activated by σ^S^ at the RNA level, suggesting an inverse correlation between regulation by σ^S^ at the protein and RNA levels. Most interestingly, these unexpected regulatory patterns were observed independently of the FDR value. These findings suggest that the observed changes at the protein level were the result of post-transcriptional regulatory effects by σ^S^. It is also possible that some genes were selected in the proteomics approach, and not by RNA-seq, due to differences in the sensitivity of the two methods.

Overall, the proteomics data confirmed the positive effect of σ^S^ on transcription of genes involved in central energy metabolism (glycolysis and the pentose phosphate pathway, mixed acid fermentation), glycogen and trehalose metabolism, arginine degradation, putrescine synthesis and degradation, and antioxidant pathways (catalases, superoxide dismutase, glutaredoxins, ferritins and Fe-S repair proteins) (see details in ref. 16 and Supplementary Dataset S3). Genes down-regulated by σ^S^ at both the protein and RNA levels encode porins, TCA cycle enzymes and proteins of unknown functions^16, 17^ (Supplementary Dataset S3).

### Regulatory effects of σ^S^ at the RNA level, not transferred to the protein level

The abundance of 1504 proteins was found unchanged in the Δ*rpoS* mutant, with respect to the wild type strain (Fig. 2a, Supplementary Dataset S3). Surprisingly, a fraction of the corresponding genes (309 genes, Fig. 2d) was differentially expressed in RNAseq experiments, among which 135 showed highly significant regulation^16^ (p < 0.001, Supplementary Dataset S3). Thus, for these genes, σ^S^ regulation at the RNA level was not transferred to the protein level. This regulatory profile was similar to that of STM14_5292 (*ytfK*), one of the small genes strongly activated by σ^S^ at the RNA level^16^ (Supplementary Table S1) but not at the steady state level of the protein (Fig. 1b). Consistent with these data, the Δ*rpoS* mutation strongly impaired expression of a transcriptional STM14_5292-*lacZ* fusion, but had a minor (and positive) impact on the expression of the translational STM14_5292-*lacZ* fusion (Fig. 1c). The LacZ protein was fused at the C- terminus of YtfK to account for translation and turnover of the protein. Since protein stability usually exceeds transcript stability in bacteria^38^, σ^S^ activation of STM14_5292 transcription might be masked by a long half-life of YtfK produced during the exponential phase of growth (Fig. 1b). Alternatively, translation and/or stability of the protein might be improved in the absence of σ^S^, compensating the defect in gene transcription. More generally, it is possible that, in some cases, transcription activation by σ^S^ contributes to proteostasis by compensating for impaired protein translation or stability in stationary phase, due to accumulation of σ^S^ itself or to other signals/regulators. Alternatively, when σ^S^ function is restricted, other signals/regulators may be activated to override at the protein level a decrease in transcription of a number of σ^S^-dependent genes.

**Figure 3:**
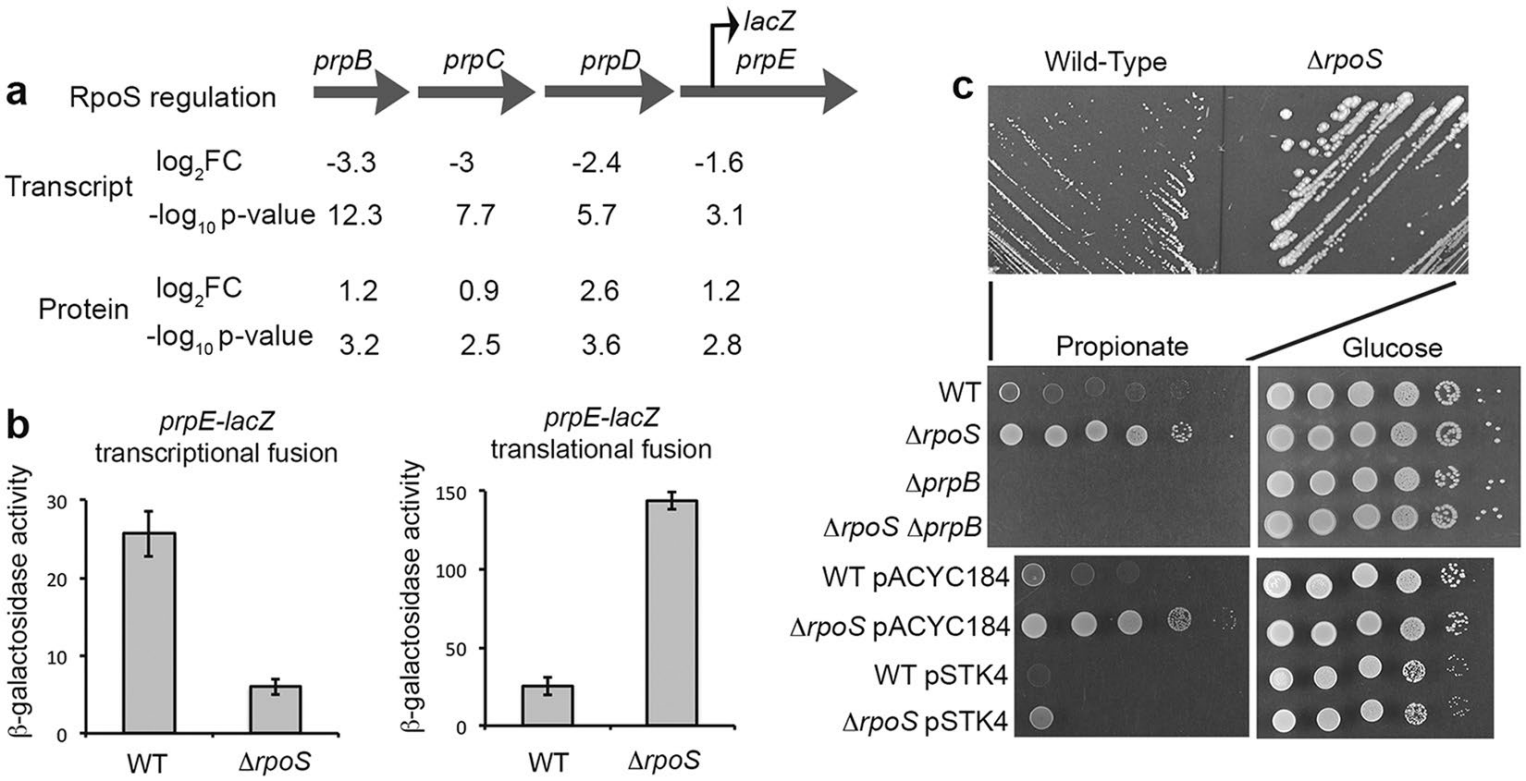
Regulation of the *prpBCDE* genes and propionate utilization by σ^S^. (**a**) Schematic representation of the *prpBCDE* operon and its regulation by σ^S^ at the transcript^16^ and protein levels (Supplementary Dataset S3). A broken arrow indicates the position of the transcriptional and translational *lacZ* fusions in the *prpE* gene. (**b**) Expression of the transcriptional and translational *prpE*-*lacZ* fusions in the wild type and Δ*rpoS* strains, grown for 18 h in LB at 37 °C. Bar graphs represent the mean β-galactosidase activity, and error bars represent standard deviation of at least three independent experiments. (**c**) Effect of the Δ*rpoS* and Δ*prpB* mutations on *Salmonella* growth at the expense of propionate and glucose as sole carbon source. Growth of the wild type *Salmonella* strain (VF6910) and its Δ*rpoS* (VF8158), Δ*prpB* (VF7985) and Δ*rpoS*Δ*prpB* (VFC47) derivatives was assessed on minimal medium with propionate or glucose (20 mM). Stationary phase cultures in LB were washed, resuspended in phosphate-buffered saline (PBS)^54^ to OD_600_ of 1.0, and 5 μl of serial dilutions were spotted onto plates that were incubated at 37 °C (24 h and 72 h for glucose and propionate utilization, respectively). Empty vector pACYC184 and plasmid pSTK4 carrying the *rpoS* gene were used in complementation experiments.

**Figure 4:**
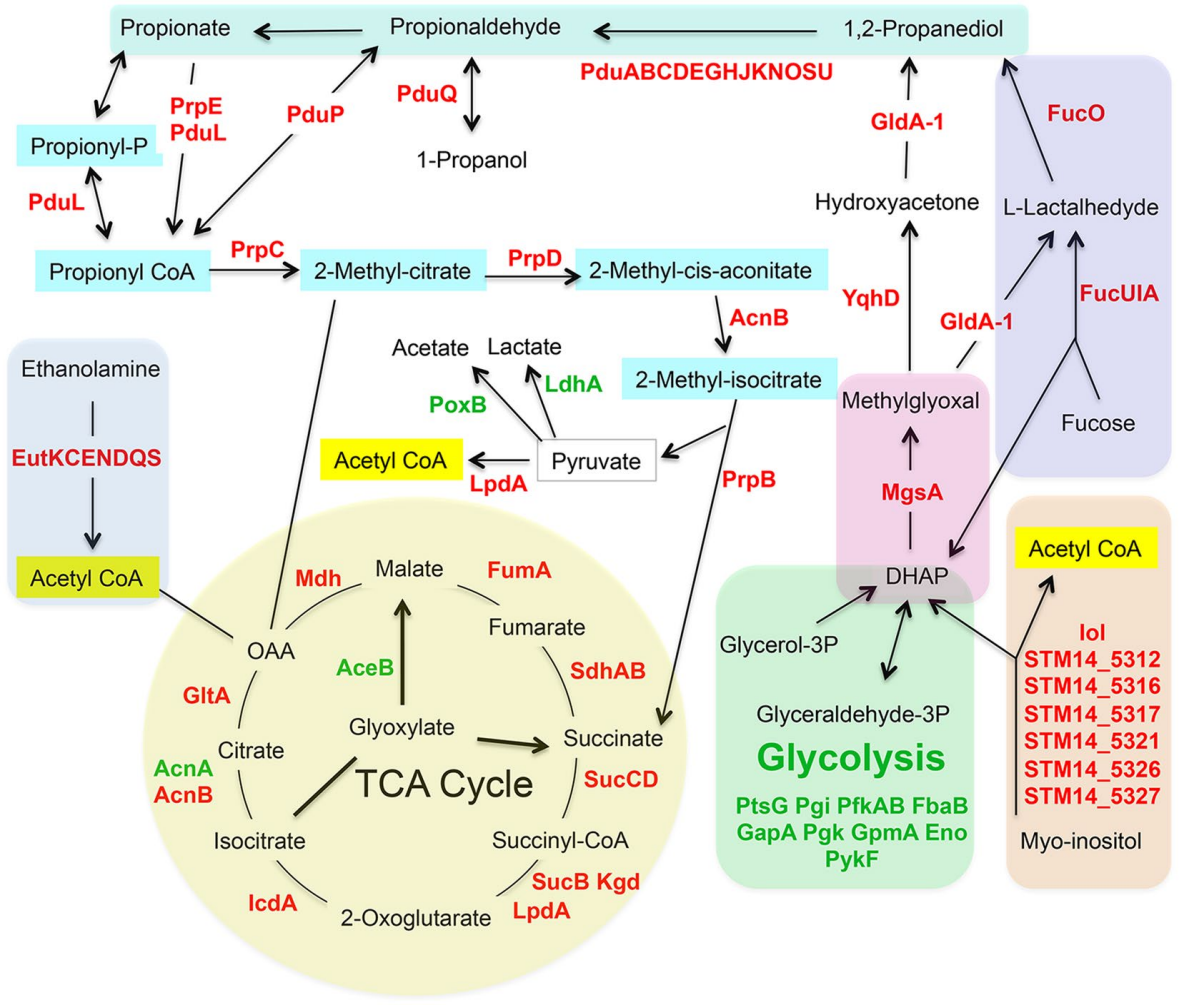
σ^S^ control of metabolic pathways revealed by proteomic analyses. Metabolic pathways down-regulated by σ^S^ are shown schematically, including the TCA cycle, propionate catabolism (Fig. 3) and myo-inositol, ethanolamine, propanediol and L-fucose degradation (Fig. 5). The ability of *S*. Typhimurium ATCC14028 to utilize myo-inositol is conferred by the *iol* genes (from STM14_ 5307/*iolR* to STM14_5327/*iolH*)^47, 62^. The *pduABCDEFGHJKLMNOPQSTUVWX* and *eutSPQTMNEJGHABCLKR* gene clusters allow *Salmonella* to grow using propanediol and ethanolamine as sole carbon source, respectively^63–66^. Proteins involved in the indicated pathways and showing differential abundance in the wild type and Δ*rpoS* strains of *Salmonella*, with a p-value of less than 0.05, are indicated (Supplementary Dataset S3). Proteins in red and green were down- and up-regulated by σ^S^, respectively. DHAP: Dihydroacetone phosphate.

Interestingly, a few genes, such as the *prpBCDE* genes, showed antagonistic regulation by σ^S^ at the RNA and protein levels (Fig. 3a). The *prpBCDE* operon encodes enzymes of the methyl citrate cycle involved in propionate catabolism^39^ (Fig. 4). The *prp* genes were activated by σ^S^ at the RNA level^16^ and down-regulated at the protein level (Fig. 3a). Consistent with these data, σ^S^ activated a transcriptional *prpE*-*lacZ* fusion, but had a negative impact on the translational *prpE*-*lacZ* fusion (Fig. 3b). The negative effect of σ^S^ on the abundance of the Prp proteins is consistent with the higher capability of the Δ*rpoS* mutant, compared to the wild type strain, to growth on propionate as a sole carbon source^40^ (Fig. 3c). As expected, a *prpB* mutation prevented the growth of the Δ*rpoS* mutant, and the *rpoS* gene on pSTK4 complemented the Δ*rpoS* mutation (Fig. 3c). In *E*. *coli* K12, the *prp* genes are also positively controlled by σ^S^ at the RNA level^13^. However, since *rpoS* disruption did not improve *E*. *coli* K12 growth on propionate^41^, *prp* regulation may be different in *E*. *coli* and *Salmonella*. This may be related to the finding that the *prp* operon of *E*. *coli*, in contrast to that of *S*. Typhimurium, is interrupted by repetitive elements (Supplementary Fig. S8 and references therein).

The complex regulation of the *prp* genes by σ^S^ in *Salmonella* might account for the dual effects of propionate catabolism on bacterial growth^39^. Short chain fatty acids, like propionate, have antibacterial activities and are used as preservatives in food industry^39^. *Salmonella* may be frequently exposed to propionate in the anaerobic environment of the gut, this exposure reducing its capacity to invade and colonize intestinal epithelial cells^42^. Thus, in such environment, activation of propionate catabolism may provide a competitive advantage to *Salmonella* ^42^. However, catabolism of propionate is a risk because 2-methylcitrate is a potent inhibitor of cell growth^39^. *Salmonella* may thus be under constant pressure to maintain levels of both propionate and 2-methylcitrate low enough to avoid the negative effects caused by accumulation of these compounds. Under the aerobic conditions used here, where the activity of the TCA cycle is down-regulated by σ^S^, excessive propionate catabolism may have an inhibitory effect due to accumulation of 2-methylcitrate (Fig. 4). Lowering abundance of the Prp proteins under these conditions may thus confer a fitness advantage. A dual and antagonistic control of *prp* expression would allow σ^S^ to monitor balanced Prp expression, and to shift rapidly the balance according to the cell needs under changing environmental conditions.

### Novel physiological effects of σ^S^ revealed by proteomic analyses

**Figure 5:**
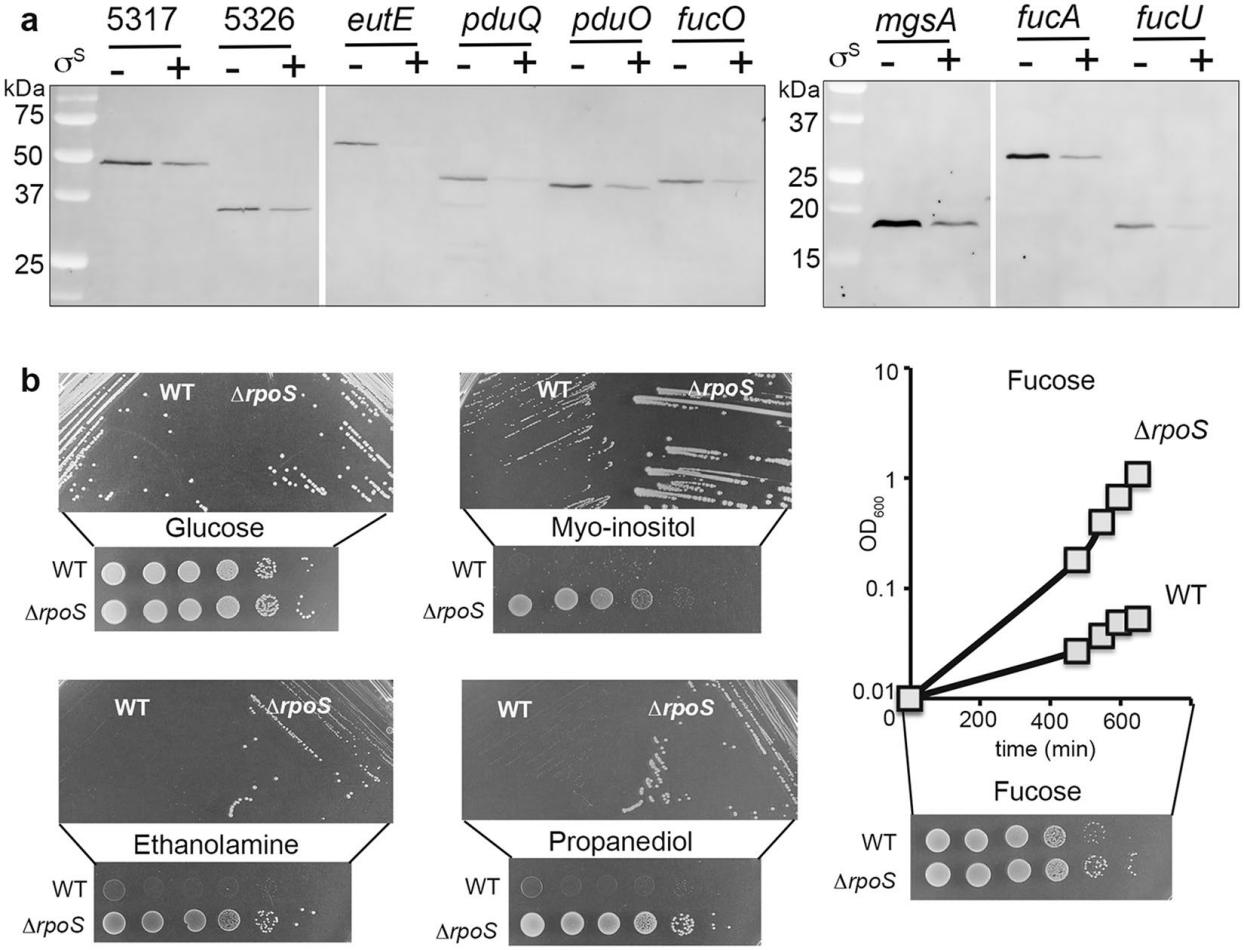
Physiological validation of σ^S^ down-regulation of metabolic functions revealed by proteomic analyses. (**a**) Immunodetection of 3xFlag-tagged proteins of interest with an anti-Flag antibody in stationary phase (OD_600_ of 4) LB cultures of the wild type and *rpoS* strains (VF6910 and VFC331). The amount of proteins in whole-cell lysates was determined and 20 μg of total proteins were loaded in each slot. Reversible Ponceau staining of the membrane was used to check proteins transfer. Similar results were obtained in at least two independent experiments. (**b**) Effect of a Δ*rpoS* mutation on *Salmonella* growth at the expense of glucose, myo-inositol, propanediol, ethanolamine and L-fucose as sole carbon source. For growth assays on plates, stationary phase cultures in LB of the wild type (WT, VF6910) and Δ*rpoS* (VF8158) strains were washed and spotted onto plates as indicated in Fig. 3. The bacterial suspensions were also streaked on plates to assess colony size. The bacterial growth of the wild-type strain and Δ*rpoS* mutant was also compared in liquid medium containing L-fucose. Stationary phase cultures in LB were washed, resuspended in PBS^54^ to an OD_600_ of 1.0, and bacteria were inoculated to minimal medium containing L-fucose at an initial OD_600_ of 0.01. The growth phase was determined by measuring culture turbidity at an optical density of 600 nm.

Among genes negatively controlled by σ^S^ at the protein level, we noticed several products of the *iol*, *pdu*, *eut* and *fuc* genes, involved in the catabolism of myo-inositol, propanediol, ethanolamine and L-fucose, respectively (Fig. 4 and Supplementary Dataset S3). Western blot analysis of relative levels, in the Δ*rpoS* and wild type strains, of products of the *fuc*, *iol*, *pdu* and *eut* genes validated the proteomics data (Fig. 5a). Also consistent with these results, the Δ*rpoS* mutant grew better than the wild type strain at the expense of myo-inositol, fucose, propanediol and ethanolamine as sole carbon sources (Fig. 5b) and the Δ*rpoS* mutation was complemented by a plasmid carrying *rpoS* (Supplementary Fig. S9). Production of the MgsA protein that converts dihydroacetone phosphate (DHAP), a product of myo-inositol and fucose degradation, into methylglyoxylate was also down-regulated by σ^S^ (Figs 4 and 5a and Supplementary Dataset S3).

**Figure 6:**
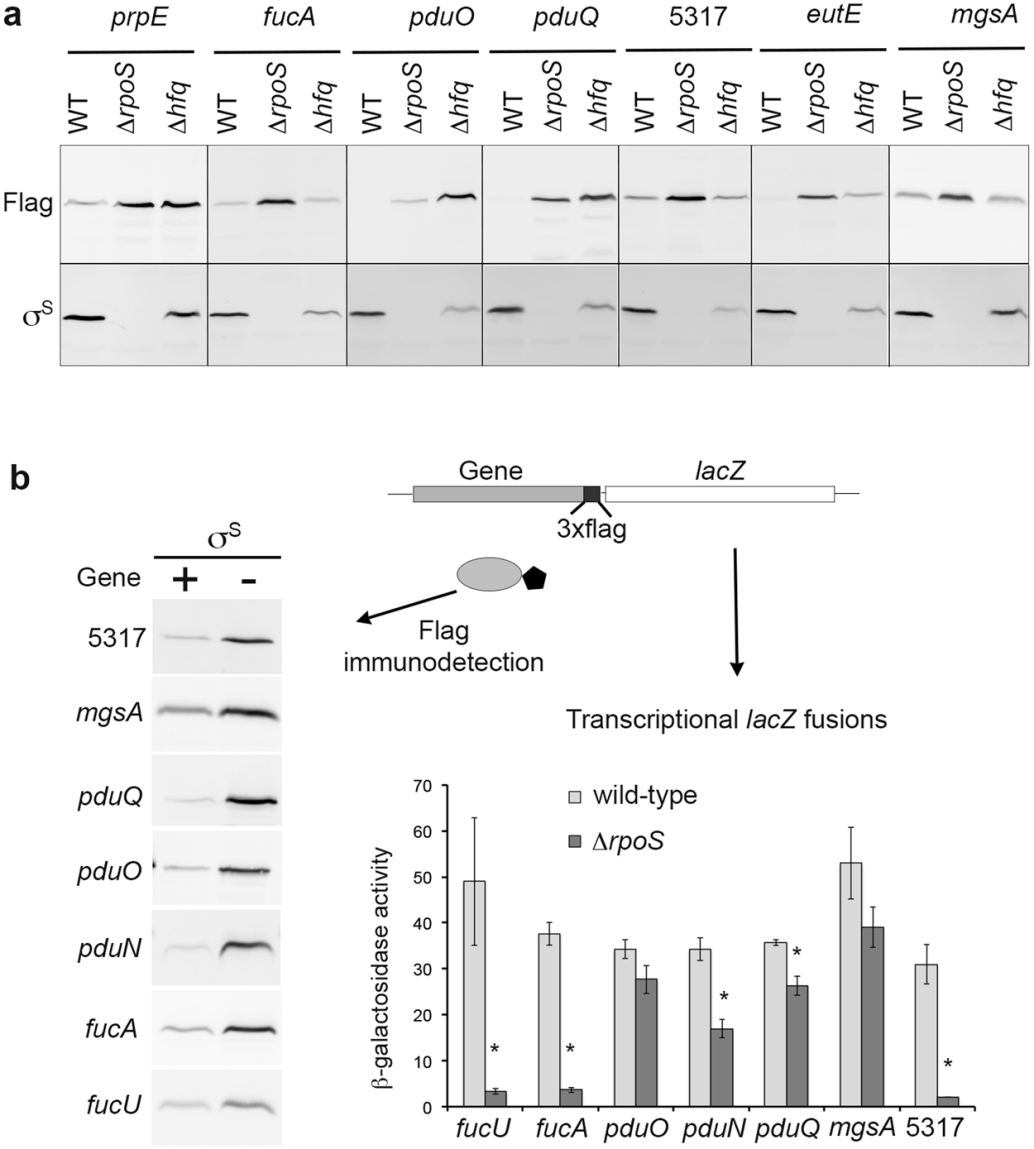
Post-transcriptional down-regulation of genes involved in metabolic functions. (**a**) Immunodetection of the 3xFlag-tagged proteins of interest was performed with an anti-Flag antibody in stationary phase (OD_600_ of 4) LB cultures of the wild type, Δ*rpoS*, and Δ*hfq* strains (VF6910, VFC331 and VFA714, respectively). The amount of proteins in whole-cell lysates was determined and 20 μg of total proteins were loaded in each slot. Reversible Ponceau staining of the membrane was used to check proteins transfer. Coomassie blue–stained gels of identical protein samples are shown in Supplementary Fig. S13. (**b**) Schematic representation of constructs used to assess expression of transcriptional *lacZ* fusions in the indicated genes, and production of the corresponding 3xFlag-tagged proteins, in stationary phase (OD_600_ of 4) LB cultures the wild type and Δ*rpoS* strains (VF6910 and VFC331, respectively). Left: Immunodetection of 3xFlag-tagged proteins. The amount of proteins in whole-cell lysates was determined and 20 μg of total proteins were loaded in each slot. Reversible Ponceau staining of the membrane was used to check proteins transfer. Coomassie blue–stained gels of identical protein samples are shown in Supplementary Fig. S13. Right: β-galactosidase assays. Bar graphs represent the mean β-galactosidase activity, and error bars represent standard deviation of at least three independent experiments. *β-galactosidase activity in the Δ*rpoS* mutant significantly different from that in the wild-type strain (p < 0.05, two-tailed t test).

To our knowledge, this is the first report of positive effects of a Δ*rpoS* mutation on catabolism of myo-inositol, L-fucose, ethanolamine and propanediol. Transcript levels of the corresponding genes were not significantly affected by the Δ*rpoS* mutation^16^ (Supplementary Dataset S3), suggesting that σ^S^ down-regulates production of the gene products post-transcriptionally. The underlying molecular mechanisms are unknown and will be the subject of more detailed studies. Interestingly, the *Salmonella prp* and *pdu* genes are down-regulated, at the protein level only, by the RNA-binding protein Hfq^43^ (Fig. 6a). Increased abundance of the EutE protein was also observed in the *Salmonella* Δ*hfq* mutant, compared to the wild type (Fig. 6a). It is thus possible that σ^S^ down-regulates expression of the *prp*, *pdu* and *eut* genes using σ^S^-dependent sRNAs^16, 28^, together with Hfq. However, since Hfq favours *rpoS* translation^2, 3, 44^ (Fig. 6a), effects of Hfq on expression of these genes may be indirect *via* σ^S^ regulation. An effect of Hfq *via* σ^S^ would be consistent with the observed activation by Hfq of several σ^S^-activated genes^43, 44^ (Supplementary Table S1).

As mentioned above, transcript levels of the *pdu*, *iol*, *eut*, *fuc*, and *mgsA* genes were not significantly increased in the Δ*rpoS* strain, compared to the wild type strain^16^. However, their low transcript abundances (Supplementary Dataset S3) might have excluded them from the list of σ^S^-activated genes during the comparative transcriptomic analysis^16^. To address this issue, regulation of some of these genes was assessed in strains into which *lacZ* was inserted downstream of the flag-tagged genes to create transcriptional fusions (Fig. 6b). The *lacZ* insertion did not affect σ^S^ regulation of the flag-tagged proteins (compare data in Figs 5a and 6b). No or moderate effects of the Δ*rpoS* mutation were observed on the expression of *lacZ* fusions in the *mgsA* and *pdu* genes (Fig. 6b). In sharp contrast, expression levels of *lacZ* fusions in the *fucU*, *fucA* and STM14_5317 genes were strongly decreased in the Δ*rpoS* mutant, compared to the wild type strain, indicating that these genes belong to the class of genes showing antagonistic regulation by σ^S^. These data suggest that the number of genes transcriptionally activated by σ^S^ might have been under-estimated in the RNA-sequencing analysis for genes with low transcript abundances^16^. As a consequence, the percentage of genes showing antagonistic regulation by σ^S^ might exceed 5% (Fig. 2c). Considering the extensive overlapping between σ factor binding sites and the similarity in the −10 box sequence motifs of σ^70^ and σ^S^ promoters^3, 9, 14^, it is possible that, in stationary phase, σ^S^ provides basal transcript levels for genes otherwise transcribed by other σ, while controlling their expression post-transcriptionally.

Like propionate, ethanolamine, propanediol, L-fucose and myo-inositol are abundant compounds in food and in anaerobic environments of the gut, and their utilization may confer an advantage to *Salmonella* within natural environments^45–47^. It is noteworthy that myo-inositol catabolism confers a fitness advantage to *Legionella pneumophila* in macrophages and amoebae, and that σ^S^ has a positive effect on expression of the *iol* operon and myo-inositol uptake^48^. It is possible that expression of the *Salmonella prp*, *iol* and *fuc* genes is activated *in vivo* by σ^S^ and provides fitness advantages to the pathogen. However, as hypothesized above for the *prp* genes, in the growth conditions used in the present study, the amount of the *fuc*, *pdu*, *iol*, and *eut* products may be adjusted according to the activity of the TCA cycle (Fig. 4). In addition, catabolism of myo-inositol and L-fucose produces DHAP that can be converted by the MgsA protein into methylglyoxal, a cytotoxic and mutagenic product affecting DNA and proteins^49^. DHAP can also be produced from glyceralhedyde-3 phosphate *via* excessive carbon flux through glycolysis. Since σ^S^ activates expression of genes in the glycolytic pathway (Supplementary Dataset S3 and ref. 16), an increased carbon flux in the first half of glycolysis, and myo-inositol and L-fucose degradation, might give rise to excess DHAP and its conversion to methylglyoxal by MgsA, a process detrimental for the cell. Therefore, down-regulation of *mgsA* by σ^S^ might confer a fitness advantage to the cell.

From the few examples discussed here, it is tempting to speculate that a number of negative effects of σ^S^ on protein production, in the growth conditions used, aim at buffering toxic effects that metabolic rewiring by σ^S^ might generate in stationary phase cells. Since several genes appear to be down-regulated by σ^S^ only or mainly at the protein level in *Salmonella* (Supplementary Dataset S3), their study might pinpoint to yet uncovered σ^S^ post-transcriptional mechanisms maintaining the delicate balance between cellular preservation during quiescence and re-growth potential, under various conditions.

## Conclusion

Our global proteomic analyses provide new clues about physiological and regulatory mechanisms controlled by σ^S^ in non-actively growing *Salmonella*. σ^S^, directly or indirectly, modulates the expression of 38% of the observed *S*. Typhimurium proteome, including a broad spectrum of *Salmonella* proteins needed for various biological processes, and proteins that have not been functionally characterized. Western blotting to a number of proteins and growth assays have validated these results.

One part of the study focused more specifically on σ^S^-dependent sRNAs coding for uncharacterized small proteins that might play as-yet-unidentified roles in *Salmonella* fitness during the quiescence phase. Our data suggest that a number of annotated sRNAs identified in global transcriptome analyses are coding sRNAs, notably STnc1330 and probably also IsrI and STnc1110. Some coding sRNAs, such as IsrI, associate with Hfq^44^ (Supplementary Table S1) and might thus have a dual function, as small and messenger RNA.

Study of small proteins is an emerging research topic in bacteria and eukaryotic cells^50^. Due to their small size, these proteins usually act by modifying the activity of larger proteins/complexes or RNAs, *via* physical interactions with them. A fraction of the small σ^S^-dependent proteins identified here could interact with membrane proteins or complexes, and modulate important features of the membrane, such as permeability and transport, or have a stabilizing role, contributing to the known role of *rpoS* in membrane resilience in stationary phase^51^. For their part, small soluble σ^S^-dependent proteins could act as chaperones, facilitate protein synthesis or protein degradation and autophagy upon stress exposure, or they may have a toxic function. The main function of these small proteins may be to act as regulators, to increase plasticity and dynamics of adaptive functions. Such “modulatory activities” might be difficult to unravel, which could explain why a very few number of small proteins have been functionally characterized so far. In addition, some proteins may have partially redundant functions, such as those encoded by the paralogous genes identified in this study, which might complicate the phenotypic analyses of mutants. Identification of potential biological interactions involving small σ^S^-dependent proteins in future studies could provide insights into their functions.

Until recently, the σ^S^ response was believed to be predominantly transcriptional. In this study, direct dependency for gene expression between transcript levels and protein levels was observed for a large majority of proteins up-regulated by σ^S^, while the majority of genes encoding down-regulated proteins showed no changes in mRNA levels. The observation that only 20–25% of protein changes in this category can be matched to significant transcriptional changes was unexpected, but can be explained by altered translation or turnover rates of proteins, irrespective of mRNA levels. Such modifications can result from σ^S^-dependent regulatory mechanisms, involving proteins with metabolic functions, RNA-binding or chaperones-like proteins, and regulatory sRNAs controlled by σ^S^ ^16, 28^. Additionally, the lack of functional σ^S^ protein likely favours the accumulation of cellular damages, such as mis-folded proteins, and might reduce the efficiency of protein turnover or recycling/autophagy mechanisms, resulting in an increase in the abundance of some proteins in the Δ*rpoS* mutant.

Post-transcriptional regulation would allow σ^S^ to rapidly decrease expression of a subset of genes by actively destroying the mRNAs or proteins, which may be particularly relevant for genes with long mRNA or protein half-lives. The fast reversibility of post-transcriptional mechanisms would confer to the cell the ability to rapidly fit its physiology to changing conditions. Post-transcriptional regulatory mechanisms might endow σ^S^ with repressor functions for fine-tuning expression of target genes for which transcription rates are higher in the presence of σ^S^ (for example the *prp*, *iol*, and *fuc* genes, Figs 3 and 6). Such dual and antagonistic regulatory circuits might be well adapted to control expression of genes showing antagonistic phenotypic pleiotropy (*i*.*e*. their expression provides a fitness advantage in some environmental conditions and a fitness cost in others). They might be pivotal to favour cell survival in stationary phase and rapid outgrowth from dormancy under varying conditions. The complexity of the σ^S^ effects probably fits the cell physiology to the trade-off between cellular maintenance during the quiescence state and re-growth potential. Though challenging questions remain open, this work provides solid basis for deeper exploration of novel regulatory and physiological aspects in quiescent cells.

## Methods

### Bacterial strains, bacteriophage, plasmids and growth conditions

Strains and plasmids are listed in Supplementary Table S2. Bacteriophage P22HT105/1*int* was used to transfer mutations and *lacZ* fusions between *Salmonella* strains by transduction^52^. Green plates, for screening for P22-infected cells or lysogens, were prepared as described previously^53^. Bacteria were routinely grown in Luria-Bertani medium (LB)^54^ at 37 °C under aeration. M63 minimal medium^55^ was used to assess *Salmonella* growth at the expense of various carbon sources: glucose (20 mM), propionate (20 mM), L-fucose (25 mM), myo-inositol (55.5 mM), ethanolamine (25 mM) and propanediol (25 mM). For growth on propanediol and ethanolamine, the medium was supplemented with cobalamine (200 nM). For growth assays on plates, stationary phase cultures in LB were washed, resuspended in phosphate-buffered saline (PBS)^54^ to OD_600_ of 1.0, and 5 μl of serial dilutions were spotted onto plates that were incubated at 37 °C. Antibiotics were used at the following concentrations (in μg per ml): carbenicillin (Cb), 100; chloramphenicol (Cm), 15 for the chromosomal resistance gene and 30 for the plasmid resistance gene; kanamycin, (Km) 50; and tetracycline (Tet) 20.

### Global proteomics analyses

#### Bacterial culture

Experiments were performed in biological triplicates. The wild type and Δ*rpoS Salmonella* strains (VF6910 and VFC331, respectively) were grown in LB at 37 °C for 18 h. Fifty ml of cultures were centrifuged and pellets were resuspended in 10 ml Tris-HCl 100 mM (pH 7.4), Urea 8 M. The cell suspension was lysed at 4 °C by a Cell disrupter (Constant System Ltd), centrifuged for 15 min at 4 °C and 4,500 rpm, and the soluble fraction was immediately freezed in liquid nitrogen and stored at −80 °C. The amount of proteins in the cell lysates was determined using the DC Protein Assay kit (Bio-Rad). Integrity of the samples was checked by SDS-PAGE. This was done by resolving 10 μg of all lysates on an SDS-polyacrylamide gel, followed by Coomassie staining. Absence of protein degradation and uniform intensity of major bands across all lysates were considered as indicators of sample integrity and accuracy of protein quantification.

#### Protein digestion

Cell lysates were sonicated 2 × 1 min on ice with a Hielscher Ultrasound Technology UP200St equipped with the Vialtweeter Sonotrode (parameters of the AMPL mode: Amplitude 80%/Cycle 80%), centrifuged at 4 °C for 30 min at 14000 g. Subsequently, 50 μg of total proteins were reduced in 50 mM TCEP (Sigma − 646547) for 1 h, and alkylated in 50 mM iodoacetamide (Sigma - I114) for 1 h in dark. Proteins were digested with 1 μg rLys-C (Promega - V1671) for 3 h at 37 °C, and then with 1 μg of Sequencing Grade Modified Trypsin (Promega - V5111) for 16 h at 37 °C. The digestion was stop with 4% formic acid, and peptides were desalted on reversed phase C18 Sep-Pak Cartridge (Waters - WAT054955). Peptides were eluted with 2× Acetonitrile 50%/Formic acid 0.1% and 1× Acetonitrile 80%/Formic acid 0.1%. Finally, samples were dried in vacuum centrifuge and resuspended with Acetonitrile 2%/Formic acid 0.1%.

#### LC-MS/MS analysis

Online chromatography was performed with a Thermo EASY-nLC 1000 UHPLC system (Thermo Fisher Scientific, Bremen, Germany) coupled online to the Q Exactive HF instrument with a nano-electrospray ion source (Thermo Fisher Scientific). For each samples, 1 μg of peptides was injected onto a 50 cm column (EASY-Spray column, 50 cm × 75 µm ID, PepMap C18, 2 µm particles, 100 A pore size - ES803 - Thermo Fisher Scientific) and separated with a multi-step gradient from 2 to 23% acetonitrile in 135 min and 23 to 45% acetonitrile in 20 min, at a flow rate of 250 nL/min over 190 min. Column temperature was set to 50 °C. MS data were acquired using Xcalibur software, using a data-dependent top10 method with a survey scans (300–1700 m/z) at a resolution of 60,000, and a MS/MS scans (fixed first mass 100 m/z) at a resolution of 15,000. The AGC target and maximum injection time for the survey scans and the MS/MS scans were set to 3 E^6^, 100 ms and 1 E^5^, 45 ms, respectively. The isolation window was set to 1.6 m/z and normalized collision energy fixed to 28 for HCD fragmentation. We used an underfill ratio of 2.0% for an intensity threshold of 4.4 E^4^. Unassigned precursor ion charge states as well as 1, 8 and >8 charged states were rejected and peptide match was disable. Exclude isotopes was enabled and selected ions were dynamically excluded for 45 seconds.

#### Data analysis

Raw data were analysed using MaxQuant software version 1.5.3.8^56^, using the Andromeda search engine^57^. The MS/MS spectra were searched against the *Salmonella* Typhimurium strain 14028 s UniProt database containing 5,369 proteins, and against the contaminant file included in MaxQuant. The digestion mode was set to trypsin, and a maximum of two missed cleavages were allowed. N-terminal acetylation and Methionine oxidation were set to variable modifications and Cysteine Carbamidomethylation as fixed modification. Identification of protein required at least one unique peptide per protein group, and every peptide were used only once in the protein identification process by the Razor protein FDR parameter. The minimum peptide length was fixed to 7 amino acids, and the required false discovery rate was set to 1% at the peptide and protein level. The main search peptide tolerances was set to 4.5 ppm and to 20 ppm for the MS/MS match tolerance. Second peptides was enabled to identify co-fragmentation events and match between runs accepted a match time window of 0.7 min for an alignment time window of 20 min. Quantification was performed using the XIC-based LFQ algorithm, with the Fast LFQ mode as described in ref. 58. Unique and razor peptides, included modified peptides, with at least 2 ratio counts were accepted for quantification.

#### Statistical analysis

Output protein group file was integrated into Perseus^59^, the companion software of MaxQuant, to perform data filtering and statistical tests. First, contaminants, reverse identifications, and proteins only identified by site were excluded from further data analysis and a categorical annotation was applied to create two sample groups according to the two types of bacterial strain in triplicate. Second, LFQ intensities were log_2_ transformed. A protein filtering was set for the validation process, such as a protein was integrated in the final list only if the protein was identified in at least two replicates of one sample group. Third, statistical analysis of the proteome adaptation between the two bacterial strains was performed on the 2444 filtered proteins. To this effect, we decided to analyse and compare our dataset with (SI approach) and without (AI approach) missing values. Missing values for LFQ intensities were imputed and replaced by random LFQ intensities that were drawn from a normal distribution at the low detection level (Supplementary Fig. S10). Yellow indicated imputated values in Supplementary Datasets S2 and S3. In both cases, two-sided T-tests of the log_2_ transformed LFQ intensities with a permutation-based FDR calculation at 5%, 1%, 0.1% and S0 = 1^60^ were employed to determine different degrees of statistically significant proteins. This statistical process is the base of the proteomic comparison between the two bacterial strains, which is represented by the two Volcano-Plots, plotting the protein difference values against negative log_10_ transformed p-values of the two-sided T-test (Supplementary Figs S11 and S12). Proteins detected in less than two replicates of one strain and in at least two replicates of the other strain, were designated as “exclusive” to that latter strain. With the SI approach, “exclusive” proteins were considered as significant proteins (Supplementary Dataset S2). The SI approach yielded a list of 299 exclusives proteins (116 in the wild type strain and 183 in the Δ*rpoS* mutant), and three statistically significant sets of differentially abundant proteins. RpoS was found exclusively in the wild type strain, consistent with the deletion of the *rpoS* gene in the mutant, and was thus excluded from the final list of σ^S^-regulated proteins (Fig. 2). For further analyses, changes in protein abundance were considered significant only when meeting the threshold of p-value 0.05 (log_10_ > 1.3, Supplementary Dataset S3). This yielded a final list of 806 significant proteins showing differential abundance in the wild type and Δ*rpoS* strains, among which 400 proteins were selected only with a 0.5% FDR, 223 were selected with 0.5 and 0.1% FDR, and 183 were selected with 0.5, 0.1 and 0.01% FDR (Fig. 2, Supplementary Dataset S3). The AI approach, used to evaluate the significance of the 299 exclusive proteins, yielded a final list of 134 statistically significant (p-value < 0.05) exclusive proteins, including RpoS itself (Fig. 2, Supplementary Dataset S3). In total, the abundance of 939 proteins (133 exclusives and 806 differentially abundant) was regulated by σ^S^ (Fig. 2, Supplementary Dataset S3).

#### Other methods

Methods for strains construction, DNA manipulation, immunoblot analysis of proteins, enzymatic assays and sequence analyses are described in Supplementary Methods.

### Data availability statement

The mass spectrometry proteomics data have been deposited to the ProteomeXchange Consortium *via* the PRIDE^61^ partner repository with the dataset identifier PXD005256.

## Electronic supplementary material

Supplementary Information

Dataset 1

Dataset 2

Dataset 3
