# Supplementary material for: Proteome remodelling by the stress sigma factor RpoS/σS in Salmonella: identification of small proteins and evidence for post-transcriptional regulation
Source: Sci Rep. 2017 May 18;7:2127. doi: 10.1038/s41598-017-02362-3 (PMC5437024; doi:10.1038/s41598-017-02362-3)
Supplement: Supplementary file 1 — Supplementary Information [file 41598_2017_2362_MOESM1_ESM.pdf]

SUPPLEMENTARY INFORMATION

Proteome remodelling by the stress sigma factor RpoS/σ^S in *Salmonella*: identification of small proteins and evidence for post-transcriptional regulation.

Magali Lago[1,2,3§,] Véronique Monteil[1,2,4§], Thibaut Douche[5], Julien Guglielmini[6], Alexis Criscuolo[6], Corinne Maufrais[6], Mariette Matondo[5] and Françoise Norel[1,2,4*]

[1]: Institut Pasteur, Laboratoire Systèmes Macromoléculaires et Signalisation, Département de Microbiologie, rue du Dr. Roux, 75015 Paris, France

[2]: CNRS ERL6002, rue du Docteur Roux, 75015, Paris, France

[3]: Université Paris Diderot, Sorbonne Paris Cité, Cellule Pasteur, Paris, rue du Dr. Roux, 75015, Paris, France

[4]: Institut Pasteur, Unité de Biochimie des Interactions Macromoléculaires, Département de Biologie structurale et Chimie, rue du Dr. Roux, 75015 Paris, France

[5]: Institut Pasteur, Unité de Spectrométrie de Masse Structurale et Protéomique, Département de Biologie Structurale et Chimie, UMR3528, rue du Dr. Roux, 75015 Paris, France

[6]: Institut Pasteur, Bioinformatics and Biostatistics Hub, C3BI, USR 3756 IP CNRS, rue du Dr. Roux, 75015 Paris, France

§ These two authors contributed equally to this work

* Corresponding author; Email: francoise.norel@pasteur.fr Tel: (33) 140613122

Supplementary Methods

DNA manipulations, epitope tagging, *lacZ* fusions and inactivation of chromosomal genes. Standard molecular biology techniques were used[4,54]. Oligonucleotides were obtained from Sigma-Aldrich and are listed in Supplementary Table S3. DNA sequencing was performed by the Cochin sequencing platform (Paris, FRANCE). Chromosomal deletions, 3x-Flag epitope tagging, and *lacZ* fusions were generated in *Salmonella* ATCC14028 using PCR-generated linear DNA fragments (Supplementary Table S3) and λ-Red recombination-based method[16,17,67-70]. All strains were confirmed to contain the expected mutation by DNA sequencing.

Electrophoresis and immunoblot analysis of proteins. Whole-cell extracts were prepared and SDS-polyacrylamide gel electrophoresis was carried out as described[71,72]. For detection of 3x-Flag-tagged proteins during growth, exponential-phase cultures of *Salmonella* in LB at 37°C were diluted into LB prewarmed at 37°C to prolong the exponential phase, and aliquots were removed during the exponential phase and stationary phase. The amount of proteins in whole-cell lysates was determined using the DC Protein Assay kit (Bio-Rad). Equal amounts of proteins were loaded in each slot. The molecular sizes of the proteins were estimated using Precision Plus Protein Standard (Bio-Rad). Proteins were transferred to nitrocellulose blotting membranes (Amersham Protan, GE Healthcare). Reversible Ponceau staining[54] of the membrane was used to check proteins transfer. Membranes were incubated with a mouse anti-Flag antibody (F3165 Sigma) as previously described[71,72]. Bound antibodies were detected using a secondary anti-mouse antibody linked to peroxidase (A4416 Sigma) and the Pierce ECL Plus western blotting substrate (Thermoscientific).

Enzymatic assays. β-galactosidase activity was measured as described by Miller[55] and is expressed in Miller units.

Sequence analyses. Trans-membrane fragments (TM) were predicted with TMHMM (http://www.cbs.dtu.dk/services/TMHMM/). Signal peptides (SP) were predicted with SignalPep (http://www.cbs.dtu.dk/services/SignalP/). Lipoproteins (Lipo) were predicted with the LipoP 1.0 Server (http://www.cbs.dtu.dk/services/LipoP/). InterPro (https://www.ebi.ac.uk/interpro/) was used to classify proteins into families and to predict domains.

Collection of putative homologs of small σ^S-dependent genes. Each amino acid query sequence was used to perform a PSI-BLAST similarity search[73] against the NCBI protein databank (nrprot). As homology is quite difficult to assess when considering small query sequences (e.g. < 80 amino acid character states), PSI-BLAST searches were performed with relaxed parameters, i.e. word size = 2, E-value threshold = 10^5, and both substitution matrix and gap cost value adjusted according to the query lengths (for more details, see www.ncbi.nlm.nih.gov/blast/html/sub_matrix.html). This initial procedure has led to very large initial sets of candidate sequences that were next mined in order to gather those that allow observing significant similarity with the corresponding query sequence. For each set, given its position-specific score matrix (pssm) estimated during the PSI-BLAST search (for more details, see[73]), the Bit-score value was estimated against each candidate sequence and a p-value was derived by comparing this Bit-score value to those estimated against 1,000 random shuffling of the same sequence. Shuffling was performed by locally exchanging residues within a window of 20 (e.g.[74,75]). From each set, significantly similar candidates (p-value < 0.01) were therefore selected and aligned with MAFFT (version 7.273) with the L-

INS-i method[76]. These multiple sequence alignments were used to infer Hidden Markov Model (HMM) protein profiles, and final sets of putative homologs were built by performing HMM profile searches against the initial sets of candidate sequences with HMMer version 3.1b2[77] (E-value threshold = 10^{-4}). We also built a more restricted set of sequences by using an HMM E-value threshold of 10^{-5} and by filtering out outliers. We consider as outliers sequences that are too short or too long, as assessed by the modified Z-score[78].

Supplementary Figures

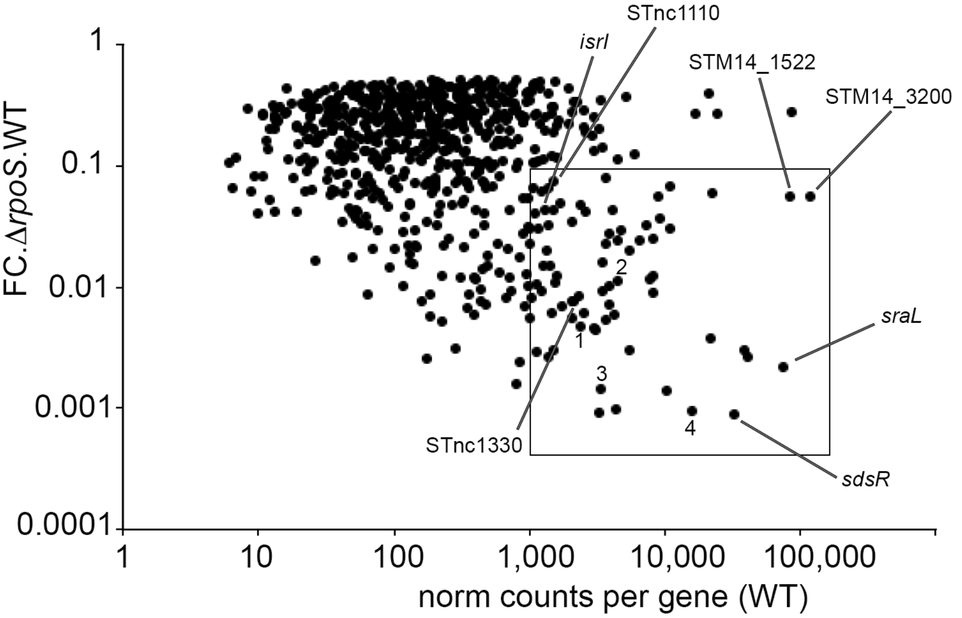

Figure S1. Relative expression level and σ^S-activation of genes identified by RNA sequencing in[16]. RNA reads, identified by RNA sequencing in the wild type and Δ*rpoS* strains of ATCC14028, were mapped to genes annotated in the genome of this strain[16]. Dots represent genes positively controlled by σ^S ($p<0.05$)[16]. The x-axis shows normalized RNA reads counts in the wild-type strain (WT), normalized to the length of the gene, thus indicating the relative expression level of the gene in the wild type strain[16]. The y-axis shows the fold change in the expression levels of the gene in the Δ*rpoS* mutant compared to the wild-type strain ($p<0.05$). Genes showing the highest read counts per gene (above 1000) and fold induction by σ^S (more than 10) are highlighted by a box. Putative arginine tRNA genes (STM14_1522 and STM14_3200) and the annotated sRNAs genes *sdsR*, *sraL, isrI*, STnc1110 and STnc1330 are labelled. RNA reads labelled dots 1-4 were assigned to the putative small ORFs STM14_0419, STM14_1274, STM14_1559 and STM14_5096 (Supplementary Fig. S4). However, analyses of the RNA reads coverage and sequence features in the corresponding genomic regions suggested that these transcripts more likely correspond to long 5’ UnTranslated Regions (UTR) of σ^S-dependent genes (see Supplementary Fig. S4 for details). The other σ^S-dependent genes in the box are listed in Supplementary Table S1. Among these, nineteen putative small ORFs of unknown function have been studied in the present work (Table 1 and Supplementary Figs. S5 and S6).

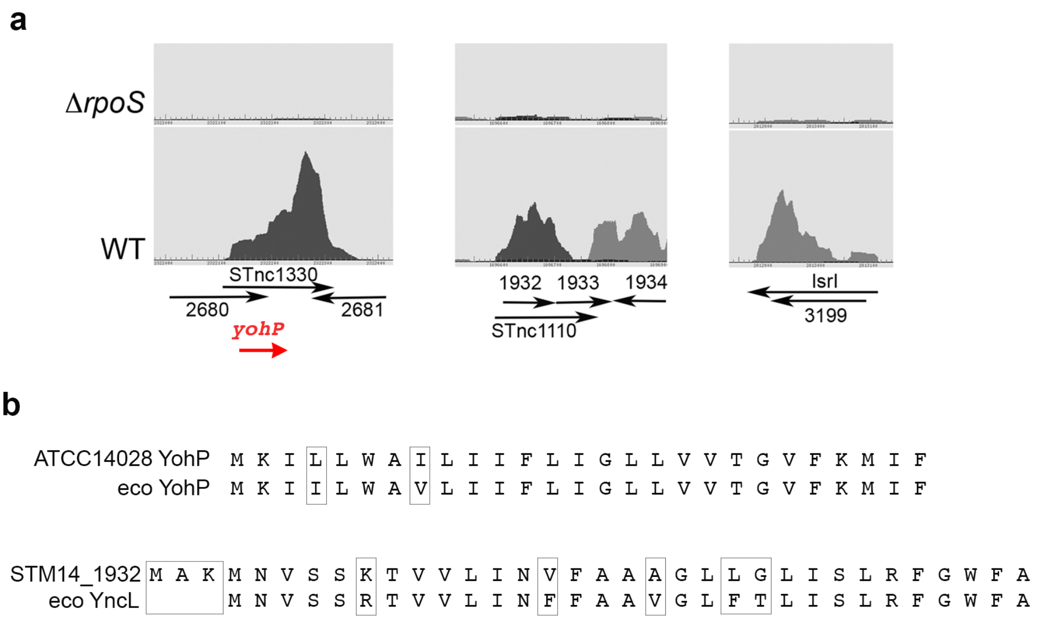

Figure S2. Sequence features of the σ^S-dependent sRNAs STnc1330, STnc1110 and IsrI. (a) RNA reads, identified by RNA sequencing in the wild type (WT) and Δ*rpoS* strains of ATCC14028, mapped to the genomic regions of STnc1330, STnc1110 and IsrI[16]. Mapped RNA reads were formatted into graph files for visualization at a strand-specific manner (black for + strand and grey for –strand) using COV2HTML[79]. The scale for RNA read counts on the y-axis is identical for the WT and Δ*rpoS* strains, but may be different from one panel to another. Annotated ORFs (indicated by the gene number in ATCC14028) and sRNAs genes are represented by arrows. STM14_2680, STM14_1932, STM14_1933 and STM14_3199, are small putative ORFs of unknown function. In this study, an additional ORF (*yohP*) was predicted in the sequence of STnc1330 and is indicated in red. (b) Predicted amino acid sequences of ORFs found in STnc1330 (*yohP*) and STnc1110 (STM14_1932) are aligned with the sequences of the YohP and YncL proteins of *E. coli* K12 (eco). The alignment of the predicted amino acid sequences of STM14_1932 and *E. coli* YncL, and the non-canonical start codon and lack of ribosome binding site for STM14_1932 (Fig. 1a), prompted us to re-annotated the start codon of this *Salmonella* ORF (Fig. 1a).

a

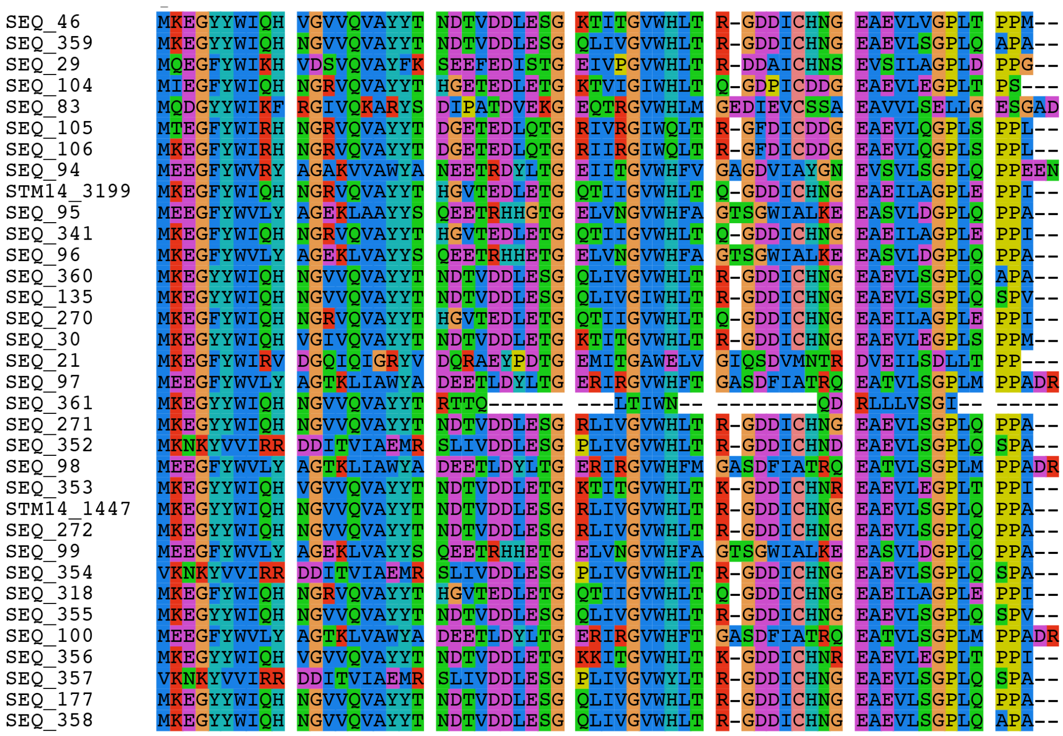

b

Id	db_prot	acc_prot	organism
SEQ_21	gb	KFC76663	*Buttiauxella agrestis* ATCC 33320
SEQ_29	ref	WP_042291610	*Citrobacter sedlakii*
SEQ_30	gb	KHE07220	*Citrobacter braakii*
SEQ_46	gb	AHY13764	*Citrobacter freundii* CFNIH1
SEQ_83	gb	EFC57535	*Enterobacter cancerogenus* ATCC 35316
SEQ_94	ref	WP_024551782	*Cronobacter helveticus*
SEQ_95	ref	WP_024551882	*Cronobacter helveticus*
SEQ_96	ref	WP_024556637	*Cronobacter pulveris*
SEQ_97	ref	WP_024558014	*Cronobacter pulveris*
SEQ_98	ref	WP_024559586	*Cronobacter pulveris*
SEQ_99	ref	WP_024560184	*Cronobacter pulveris*
SEQ_100	ref	WP_029592563	*Cronobacter pulveris*
SEQ_104	gb	KFC90312	*Leclercia adecarboxylata* ATCC 23216 = NBRC 102595
SEQ_105	gb	KFC98568	*Leclercia adecarboxylata* ATCC 23216 = NBRC 102595
SEQ_106	gb	KFC98616	*Leclercia adecarboxylata* ATCC 23216 = NBRC 102595
SEQ_135	gb	AGR59214	*Salmonella bongori* N268-08
SEQ_177	gb	ACF70051	*Salmonella enterica* subsp. *enterica* serovar Heidelberg str. SL476
SEQ_270	gb	EDX48454	*Salmonella enterica* subsp. *enterica* serovar Newport str. SL317
SEQ_271	gb	EDX48376	*Salmonella enterica* subsp. *enterica* serovar Newport str. SL317
SEQ_272	gb	EDX48942	*Salmonella enterica* subsp. *enterica* serovar Newport str. SL317
SEQ_318	ref	YP_006892401	*Salmonella enterica* subsp. *enterica* serovar Typhimurium str. LT2
SEQ_341	gb	ELX61473	*Salmonella enterica* subsp. *enterica* serovar Typhimurium str. LT2-4_delta.*ramA*::kan
STM14_3199	gb	ACY89629	*Salmonella enterica* subsp. *enterica* serovar Typhimurium str. 14028S
STM14_1447	gb	ACY87933	*Salmonella enterica* subsp. *enterica* serovar Typhimurium str. 14028S
SEQ_352	gb	EHY70819	*Salmonella enterica* subsp. *houtenae* str. ATCC BAA-1581
SEQ_353	gb	EHY70086	*Salmonella enterica* subsp. *houtenae* str. ATCC BAA-1581
SEQ_354	gb	ENZ86590	*Salmonella enterica* subsp. *houtenae* serovar 16:z4,z32:-- str. RKS3027
SEQ_355	gb	ESE91007	*Salmonella enterica* subsp. *houtenae* serovar 50:g,z51:- str. 01-0133
SEQ_356	gb	ESE85565	*Salmonella enterica* subsp. *houtenae* serovar 50:g,z51:- str. 01-0133
SEQ_357	gb	ESE89762	*Salmonella enterica* subsp. *houtenae* serovar 50:g,z51:- str. 01-0133
SEQ_358	gb	ESE81092	*Salmonella enterica* subsp. *indica* serovar 6,14,25:z10:1,(2),7 str. 1121
SEQ_359	gb	ESE82382	*Salmonella enterica* subsp. *indica* serovar 6,14,25:z10:1,(2),7 str. 1121
SEQ_360	gb	ESE83293	*Salmonella enterica* subsp. *indica* serovar 6,14,25:z10:1,(2),7 str. 1121
SEQ_361	gb	ESE64759	*Salmonella enterica* subsp. *salamae* serovar 58:l,z13,z28:z6 str. 00-0163

Figure S3. Conservation of STM14_3199. (a) Sequence alignment of the STM14_3199 product and homologous proteins. The alignment was visualized with seaview http://pbil.univ-lyon1.fr/software/seaview. (b) List of homologous sequences in other bacterial species. STM14_1447 and STM14_3199 are paralogous genes.

a

Dot	Predicted ORF	Features	Most probable assignation from RNA reads coverage and sequence analyses
1	STM14_0419	hypothetical product of 45 residues, non canonical start codon and RBS	5' end of STM14_0421 mRNA
2	STM14_1274	pseudogene (stop codon)	5' end of STM14_1275 mRNA
3	STM14_1559	hypothetical product of 58 residues, non canonical start codon and RBS	5' end of STM14_1558 mRNA
4	STM14_5096	hypothetical product of 38 residues, non canonical start codon and RBS	5' end of STM14_5097 mRNA

b

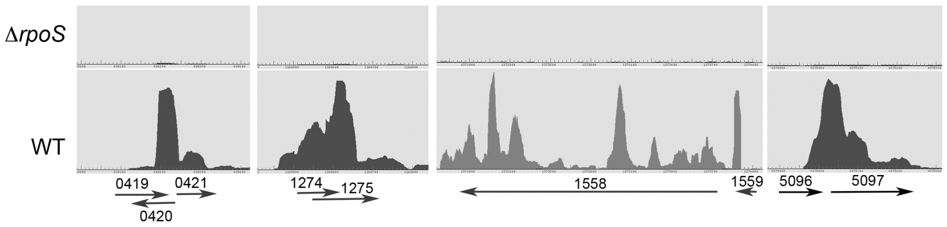

c

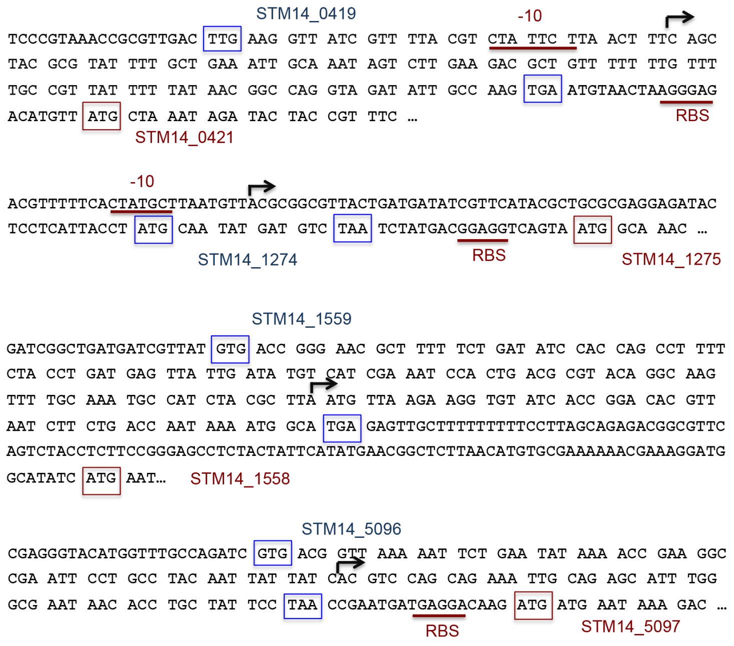

Figure S4. Long 5' UnTranslated Regions (UTR) of σ^S-dependent genes and their sequence features. (a) RNA reads labelled dots 1 to 4 on Supplementary Fig. S1 were mapped on the genome of ATCC14028 and assigned to the putative small ORFs STM14_0419, STM14_1559, STM14_5096 and the pseudogene STM14_1274[16]. However, analyses of the RNA reads coverage (b), location of transcription start sites and sequence features (c) in the corresponding genomic regions suggested that these transcripts more likely correspond to long 5' UnTranslated Region (UTR) of the σ^S-dependent genes STM14_0421, STM14_1558, STM14_5097 and STM14_1275, respectively. This hypothesis is consistent with the non-canonical start codons and lack of ribosome binding sites for the putative ORFs STM14_0419, STM14_1559 and STM14_5096 (c). (b) RNA reads, identified by RNA sequencing in the wild-type (WT) and Δ*rpoS* strains of ATCC14028 mapped to genes of interest[16] (represented by arrows and the gene number in ATCC14028). Mapped RNA reads were formatted into graph files for visualization at a strand-specific manner (black for + strand and grey for –strand) using COV2HTML[79]. The scale for read counts on the y-axis is identical for the WT and Δ*rpoS* but may be different from one panel to another. (c) DNA sequences of the 5' and upstream regions of the σ^S-dependent genes STM14_0421, STM14_1275, STM14_1558 and STM14_5097 (Supplementary Table S1) containing the putative small ORFs STM14_0419, STM14_1274, STM14_1559 and STM14_5096. Broken arrows indicate transcriptional start sites[28,37]. The -10 promoter regions and potential ribosome binding sites (RBS) are underlined. Predicted start and stop codons are indicated in boxes.

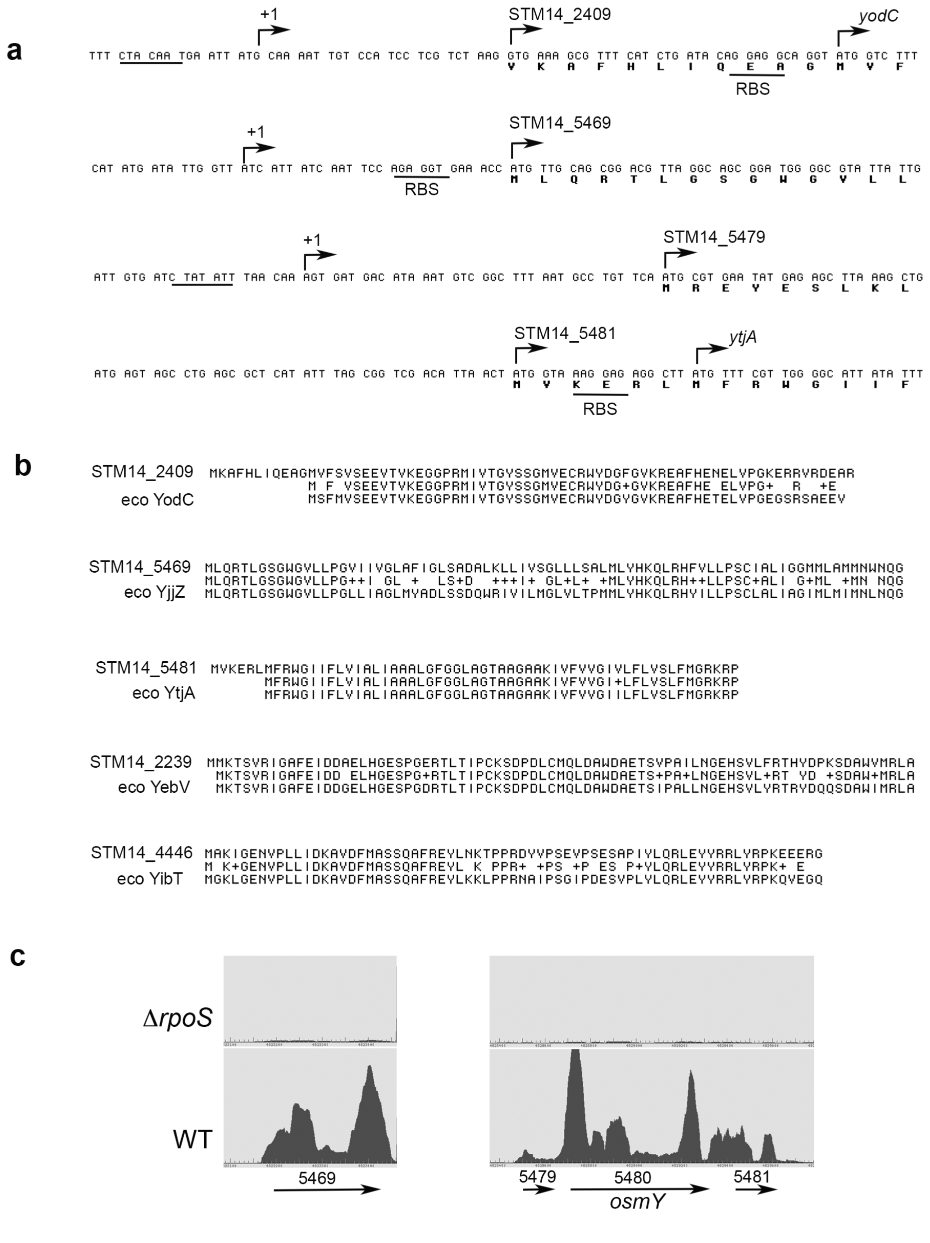

Figure S5. Sequence features of uncharacterized small ORFs activated by σ^S. (a) DNA sequences corresponding to the 5' ends and upstream regions of the σ^S-dependent small ORFs STM14_2409, STM14_5469, STM14_5479 and STM14_5481 (Table 1) are shown. Broken arrows indicate the 5' ends of ORFs and transcriptional start sites[28,37]. The -10 promoter regions and potential ribosome binding sites (RBS) are underlined. (b) Predicted amino acid sequences of the *Salmonella* σ^S-dependent small ORFs STM14_2409, STM14_5469, STM14_5481, STM14_2239 and STM14_4446 (Table 1) are aligned with that of *E. coli* K12 (eco) homologs. The translation start sites for STM14_2409/*yodC*, STM14_5481/*ytjA* and STM14_2239/*yebV* are likely located downstream of the annotated start codons (see also Supplementary Dataset S1). (c) RNA reads coverage in the STM14_5469 and STM14_5479 genomic regions. RNA reads, identified by RNA sequencing in the wild type (WT) and Δ*rpoS* strains of ATCC14028, were mapped to genes of interest[16]. The annotated genes are indicated by the gene number in ATCC14028 and represented by arrows. Mapped reads were formatted into graph files for visualization at a strand-specific manner using COV2HTML[79]. STM14_5469, STM14_5479 and STM14_5481 are small putative ORFs of unknown function. STM14_5479 is located 36 bps downstream of the promoter of *osmY* (STM14_5480)[37], a gene regulated by σ^S (Supplementary Table S1).

a

```
STM14_2091 MAEHRGGSGNFAENREKASEAGRKGGQHSGGNFKNDPQRASEAGKKGGQNSH-GGRKSDNS
STM14_1829 MAEHRGGSGNFAEDKEKASEAGRKGGQHSGGNFKNDPQRASEAGKKGGQNSHSGGRKSGN-
STM14_1275 MANHRGGSGNFAEDRERASEAGRKGGQHSGGNFKNDPQRASEAGKKGGKSSN---RNS---
```

b

**STM14_1275**	**STM14_1829**	**STM14_2091**
GGSGNFAEDR	GGSGNFAEDK	AEHRGGSGNFAENR
GGSGNFAEDRER	GGSGNFAEDKEK	GGSGNFAENR
		GGSGNFAENREK

Figure S6. Small paralogous σ^S-dependent genes. (a) Alignment of the amino acid sequences of the small paralogous genes STM14_2091/*yciG,* STM14_1829 and STM14_1275/*ymdF* (Table 1), generated with ClustalW[80]. Their products contain a KGG motif that is repeated in LEA (late embryogenesis abundant) family of proteins, playing roles as antioxidants and as membrane and protein stabilizers during extreme water stress[81]. (b) Peptides used for identification of the paralogous gene products by nLC-MS/MS.

STM14_0421	TTGACTTGAAGGTTATCGTTTTACGTCTATTCTTAACTTT
STM14_0460	TCGACGCAACGGTTACAGGGAGATGTCTATACTTAACGCT
STM14_1271	GAAATTTGCACTCTTCACAGGAGAGCATATCTTTAAATAG
STM14_1275	TAAGCGGCCTACTACGACGTTTTTCACTATGCTTAATGTT
STM14_1829	TGAATTTGACACTGCGCACAGGGCGACTAGATTTAGAACT
STM14_2091	TCAATTTGACTAATCGGTTTAACCAACTAATTTTAATAGG
STM14_2173	TTCAGAAATGCGACGCTCGACTTTGCCTATACTTAAAACG
STM14_2188	AAAAACCAAAAGTAGTGCTGTAGGGTATCTTTAATAACTA
STM14_2189	GCGCTCGCGCACGTATGGCGGCGACTCTATACTTTCAAAG
STM14_2239	TGACTGGCGCTTTTGCCTACGTTGACTACGCTGAAAAATG
STM14_2405	GCGTGTTTGCTGGTTTTCTCAACGGTCTATACTTAGGCTG
STM14_2409	ATTCGCGGTGTTTCGCACTGATTTTCTACAATGAATTATG
STM14_3597	ATCCTGGCGAATTATGTAAAGGAGGTTATGCTGAATAATG
STM14_4398	TCGCCCCCCACCCGCAAGTTTCCCGACTATTCTTAAGAGG
STM14_4446	CCTCAATGACAACGCTAAATTGATCCTAAACTCAAATAAG
STM14_5097	TATAAAACCGAAGGCCGAATTCCTGCCTACAATTATTATC
STM14_5469	TGGCTGACAAATGAGAAAATATATCATATGATATTGGTTA
STnc1110/yncL	TTATTGCGGATCTCGCCAACCCGTACTATACCCATAGGGG
STnc1330/yohP	AAAGCAGCAGATGGCTTAATACTTTCCTATACTTTGTTTG
IsrI/STM14_3199	CTAACGCCAACCTGCAATCCCAATAGCTAAACTCCTCTTA

Figure S7. Promoter sequences of σ^S-activated genes used to construct the logograph on Fig. 1d. Promoter sequences (-39 to +1) are from[28,37].

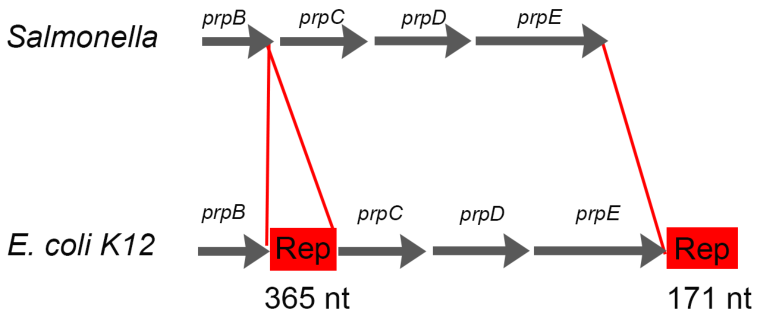

Figure S8. Schematic representation of the structure of the *prpBCDE* locus in *E. coli* K12 and *S.* Typhimurium. Compared to *S.* Typhimurium, the *E. coli* K12 *prp* operon contains additional repetitive extragenic palindromes (REP)[82,83]. The REP elements might stabilize and protect mRNA from RNAse degradation [83]. The indicated objects are not drawn to scale.

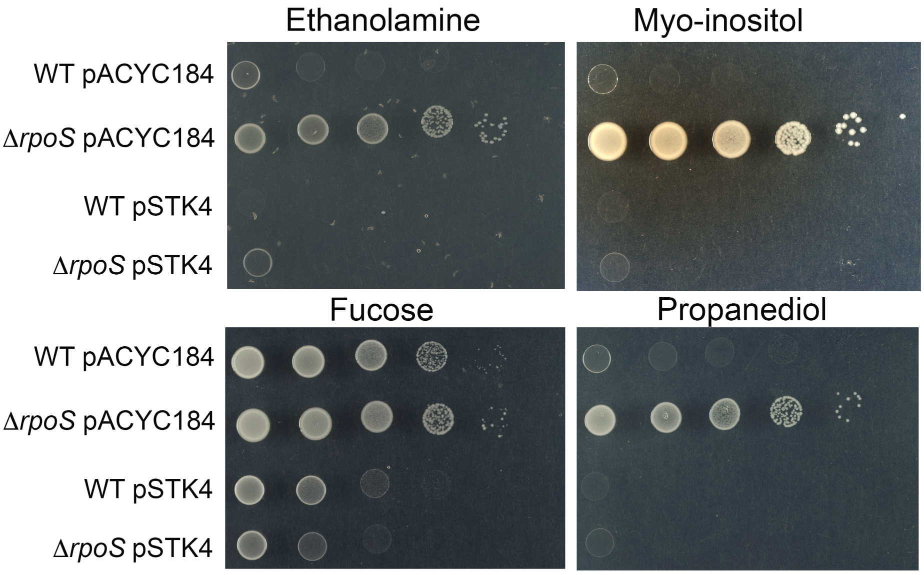

Figure S9. Complementation of the Δ*rpoS* mutation for growth at the expense of various carbon sources. Empty vector pACYC184 and plasmid pSTK4 carrying the *rpoS* gene were used in complementation experiments of the Δ*rpoS* mutation for growth at the expense of L-fucose, propanediol, ethanolamine and myo-inositol. Stationary phase cultures in LB were washed, resuspended in phosphate-buffered saline (PBS)[54] to OD_{600} of 1.0, and 5 μl of serial dilutions were spotted onto plates that were incubated at 37°C for 48 h.

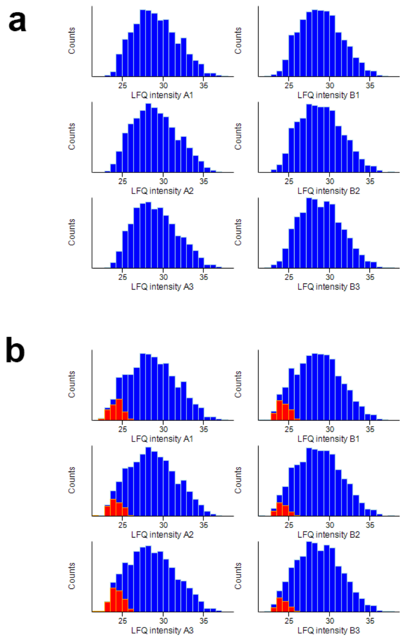

Figure S10. Histogram-Intensity distribution. Histogram of the LFQ intensity (log_2) distribution for each sample, considering the two different approaches without (a) and with (b) imputation (named respectively SI and AI approaches). Blue rods are experimental values and red rods are imputed values.

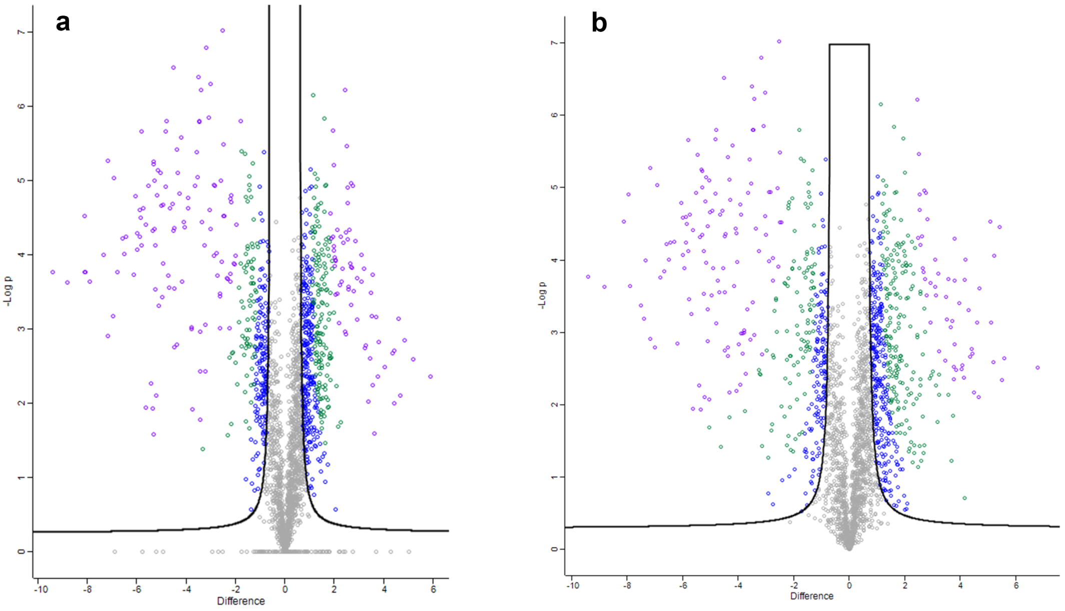

Figure S11. Volcano-Plots. Differences in LFQ intensity ($\log_2$) were plotted against negative $\log_{10}$ transformed p-values of the two-sided T-test for (a) the SI approach (without imputation) and (b) the AI approach (with imputation). Statistically significant proteins are represented by a circle colored according to the FDR value: Blue circle for FDR 5%, Green circle for FDR 1%, and purple circle for FDR 0.1%.

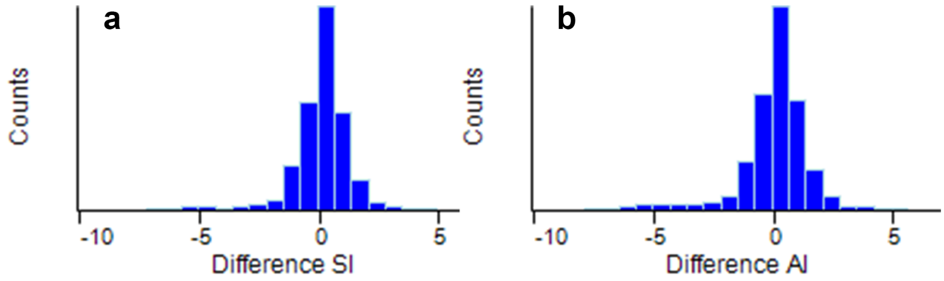

Figure S12. LFQ difference distribution. Histogram of the difference distribution for (a) the SI approach (without imputation) and (b) the AI approach (with imputation). Thereby, differences calculated from the LFQ intensities (log_2) are represented by slice in the X-axis, and the Y-axis represents the counting for each slices.

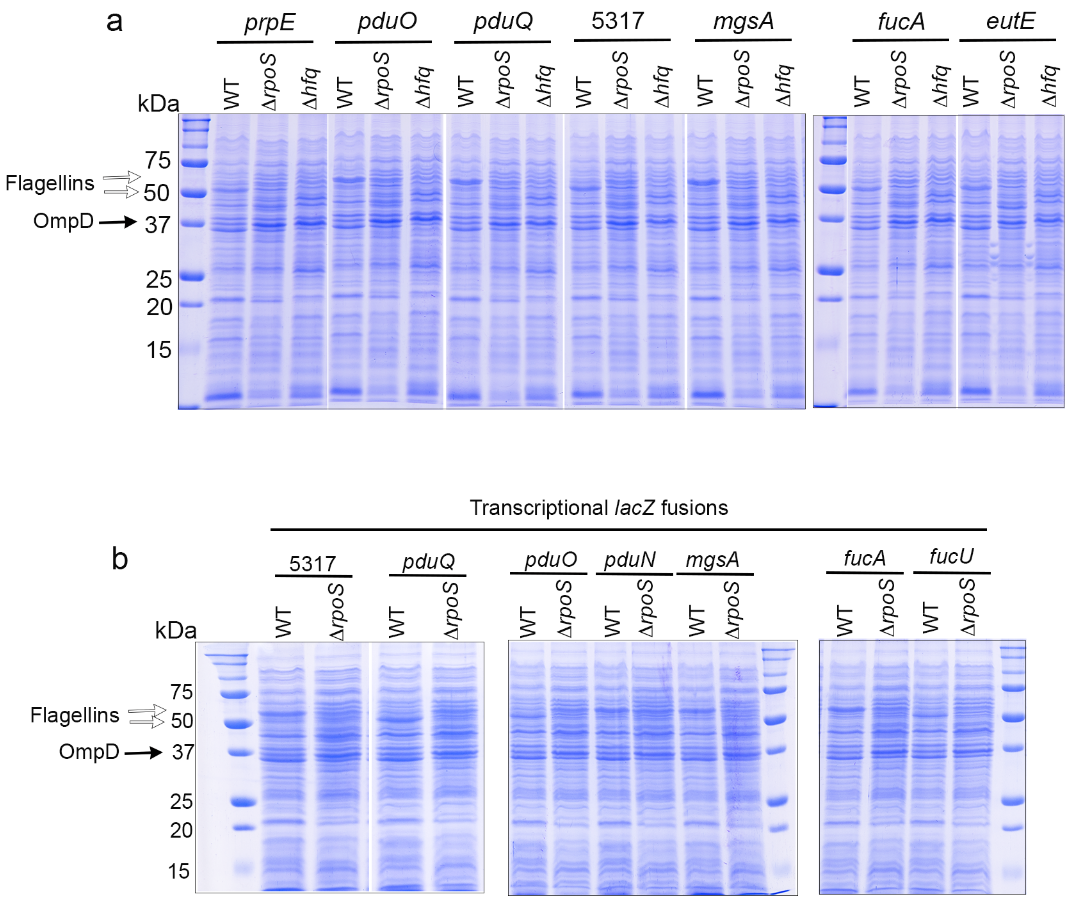

Figure S13. Coomassie Blue[54] stained gels of protein samples used for immunodetection of 3xFlag-tagged proteins (Fig. 6). (a) Effect of the Δ*hfq* and Δ*rpoS* mutations on production of Flag-tagged products of the indicated genes (Fig. 6a). (b) Effect of the Δ*rpoS* mutation on expression of transcriptional *lacZ* fusions in the indicated genes (Fig. 6b). The white arrows show the two flagellin subunits, FliC and FljB, which are alternatively expressed in *Salmonella* by a site-specific DNA inversion mechanism called flagellar phase variation. The black arrow shows the OmpD protein, which is produced to higher levels in the Δ*rpoS* mutant than the wild type strain of *Salmonella*[17].

Supplementary Tables

Table S1. Genes expressed in stationary phase under the tight control of σ^S [16] (highlighted in a box on Fig. S1)

Gene identification				RNA-seq[a]			LC-MS/MS[b]		Protein features KEGG annotation and/or Refs	Expression in *hfq* mutant[c]	Hfq Binding[44]
ATCC14028	LT2	name	length (nt)	Norm counts per gene	$\log_2$ FC	$-\log_{10}$ p-value	$\log_2$ FC	$-\log_{10}$ p-value			
STM14_0421	STM0359		102	1,371	-8.6	14.5	-5.7	4.6	Putative cytoplasmic protein	R[43,44]	
STM14_0428	STM0366	*yahO*	276	3,481	-6.0	34.3	-4.3	5.1	Periplasmic protein[84,85]	R[44]	Yes
STM14_0454	STM0384	*psiF*	321	1,117	-6.6	19.6	-5.3	5.0	Phosphate starvation inducible protein	R[43,44]	
STM14_0460	STM0389	*yaiA*	192	1,222	-4.0	6.2	Exclusive WT		Hypothetical protein	R[44]	
STM14_0527	STM0446	*bolA*	318	5,474	-5.6	33.8	-2.5	7.0	Transcriptional regulator affecting cell morphology[86]		Yes
STM14_0883	STM0759	*ybgS*	387	10,343	-9.5	62.3	-6.0	4.1	Hypothetical protein	R[43,44]	
STM14_1271	STM1118	*yccJ*	228	9,007	-4.1	11.9	-2.8	4.0	Hypothetical protein	R[43,44]	
STM14_1272	STM1119	*wrbA*	597	4,432	-5.4	32.0	-3.5	5.8	NAD(P)H:quinone oxidoreductase, TrpR binding protein[87]	R[43]	
STM14_1275	STM1121	*ymdF*	168	8,115	-6.8	13.4	-5.2	3.5	Putative cytoplasmic protein	R[43,44]	
STM14_1521	STM1261		327	1,257	-6.0	11.3	-5.5	4.9	Putative cytoplasmic protein	R[43,44]	
STM14_1558	STM1285	*yeaG*	1935	2,961	-7.8	54.8	-4.9	4.7	Serine protein kinase[85,88]	R[44]	Yes
STM14_1724	STM1427	*cfa*	1149	1,106	-4.6	24.9	Exclusive WT		Cyclopropane-fatty-acyl-phospholipid synthase[89,90]	R[43,44]	
STM14_1738	STM1440	*sodC_2*	522	1,361	-5.7	31.1	-4.5	4.3	Superoxide dismutase[91]	R[44]	
STM14_1768	STM1466	*ydgA*	1509	1,080	-4.0	19.5	-1.6	4.0	Hypothetical protein		
STM14_1829	STM1513		183	5,431	-8.4	8.4	-5.4	2.3	Putative cytoplasmic protein	R[43,44]	
STM14_1832	STM1515	*ydeI*	393	1,007	-5.0	25.0	-4.8	5.8	Involved in antimicrobial peptide resistance and virulence[92]	R[43,44]	
STM14_1886	STM1563	*osmC*	432	1,049	-6.9	35.4	-5.1	5.1	Osmotically inducible protein, oxidative stress resistance[93]	R[44]	
STM14_1924	STM1589	*yncB*	1071	1,392	-5.0	28.2	-3.9	4.8	Putative NADP-dependent oxidoreductase	R[44]	Yes
STM14_2062	STM1705	*osmB*	219	11,034	-3.9	19.2	Exclusive WT		Osmotically inducible lipoprotein[94-96]	R[43,44]	
STM14_2091	STM1728	*yciG*	183	41,351	-8.6	17.7	-5.3	1.9	Cytoplasmic protein[33-35]	R[43,44]	
STM14_2092	STM1729	*yciF*	504	4,372	-10.0	11.5	-5.6	4.4	Cytoplasmic protein[33,34]	R[43,44]	
STM14_2093	STM1730	*yciE*	507	3,307	-10.1	6.9	-5.9	4.2	Cytoplasmic protein[33,34]	R[43,44]	

STM14_2094	STM1731	*katN*	879	2,078	-7.5	5.6	-5.9	4.6	Catalase[33,34]	R[44]	Yes
STM14_2140	STM1770	*chaB*	231	2,559	-7.4	24.0	-5.3	5.3	Cation transport regulator[97]	R[43,44]	
STM14_2173	STM1797	*ymgE*	255	1,577	-6.3	32.0	ND		Putative protein	R[43,44]	
STM14_2188	STM1810		138	11,025	-5.0	23.7	Exclusive WT		Putative cytoplasmic protein	R[43]	
STM14_2189			99	7,791	-6.4	35.7	ND		Hypothetical protein		
STM14_2239	STM1851		240	2,146	-7.0	27.3	-3.3	1,4	Putative cytoplasmic protein	R[43,44]	
STM14_2405	STM1984	*yodD*	228	3,434	-6.7	28.5	-6.0	4.3	Putative cytoplasmic protein	R[44]	
STM14_2409	STM1988		219	1,363	-3.9	16.2	Exclusive WT		Putative cytoplasmic protein	R[44]	
STM14_2642	STM2141	*fbaB*	1053	1,246	-6.7	40.8	-5.3	4.4	Fructose-bisphosphate aldolase	R[44]	
STM14_2675	STM2169	*yohC*	588	3,095	-7.8	50.4	ND		Putative transport protein, membrane[98]	R[44]	
STM14_2849	STM2311	*elaB*	312	3,856	-6.6	39.6	-4.9	3.9	Inner membrane protein, binds stationary-phase ribosomes[99]	R[43,44]	
STM14_2958	STM2405		1653	1,425	-6.1	38.2	-5.1	3.3	Putative indolepyruvate decarboxylase	R[44]	
STM14_3365	STM2789	*csiD*	978	4,075	-4.5	20.7	-4.6	4.6	Carbon-starvation inducible protein		
STM14_3366	STM2790	*ygaF*	1269	1,677	-4.3	9.1	-4.4	5.4	L-2-hydroxyglutarate oxidase[100,101]		
STM14_3371	STM2795	*ygaU*	450	1,135	-8.4	18.4	-5.4	4.3	Modulation of peptidoglycan cross linking, potassium sensor[85,102,103]	R[43,44]	
STM14_3373	STM2796	*yqaE*	159	1,557	-6.5	25.6	ND		Pmp3 family membrane protein[104-106]	I [43]	Yes
STM14_3383	STM2802	*ygaM*	339	1,757	-7.2	7.3	-7.2	2.9	Inner membrane protein, binds stationary-phase ribosomes[99]	R[44]	
STM14_3458	STM2861	*sitA*	918	7,344	-5.0	29.0	-4.1	4.4	Manganese/iron transport; substrate-binding protein[107,108]		
STM14_3459	STM2862	*sitB*	822	3,637	-5.5	32.8	-5.0	4.7	Manganese/iron transport ; ATP-binding protein[107,108]		
STM14_3460	STM2863	*sitC*	861	2,037	-4.9	26.9	ND		Manganese/iron transport ; permease[107,108]		
STM14_3461	STM2864	*sitD*	849	1,156	-5.0	27.2	ND		Manganese/iron transport permease[107,108]	R[44]	
STM14_3557	STM2952	*eno*	1299	3,685	-3.7	10.3	-0.8	3.6	Phosphopyruvate hydratase		Yes
STM14_3597	STM2983	*ygdI*	228	8,208	-6.3	39.5	-5.2	4.1	Putative lipoprotein	R[44]	Yes
STM14_3896	STM3218	*oat*	1407	4,269	-7.4	51.2	-7.0	3.2	Putrescine--2-oxoglutarate aminotransferase		
STM14_3910	STM3228	*yqjC*	369	3,928	-5.2	30.0	-2.2	1.7	Hypothetical protein	R[43,44]	Yes
STM14_3911	STM3229	*yqjD*	306	2,589	-4.6	23.7	-2.6	3.5	Inner membrane protein, binds stationary-phase ribosomes[99]	R[43]	Yes
STM14_3912	STM3230	*yqjE*	399	2,367	-4.4	14.9	ND		Putative inner membrane protein	R[43,44]	Yes
STM14_3951	STM3269	*yhbO*	519	1,463	-7.4	41.9	-5.8	4.1	Glyoxalase[109-111]	R[44]	
STM14_4398	STM3648	*yiaG*	291	3,687	-7.5	45.1	Exclusive WT		Putative transcriptional regulator	R[44]	
STM14_4438	STM3680	*aldB*	1539	4,797	-5.1	31.3	-3.2	6.8	Aldehyde dehydrogenase	R[44]	Yes
STM14_4446	STM3688		210	1,004	-5.4	5.2	-2.4	4.5	Putative cytoplasmic protein	R[44]	
STM14_5097	STM4240	*yjbJ*	213	38,314	-8.4	59.9	-5.8	5.7	Putative stress-response protein	R[43,44]	Yes
STM14_5216	STM4336	*ecnB*	147	21,899	-8.0	53.9	ND		Bacteriolytic lipoprotein entericidin B[112]	R[44]	Yes
STM14_5292	STM4406	*ytfK*	207	22,625	-4.1	21.4	ND		Putative cytoplasmic protein		Yes
STM14_5430	STM4519		1371	1,510	-8.4	53.7	-5.3	5.2	Putative NAD-dependent aldehyde dehydrogenase	R[44]	
STM14_5469	STM4552		237	6,492	-5.4	30.6	ND		Putative inner membrane protein	R[43]	
STM14_5479			135	1,014	-7.5	2.9	ND		Hypothetical protein		
STM14_5480	STM4561	*osmY*	618	3,878	-7.1	46.0	-4.8	4.9	Hypothetical protein [113]	R[44]	Yes

STM14_5481	STM4562		180	2,329	-6.9	34.9	ND		Hypothetical protein	R[43]	Yes
STM14_966	STM0831	*dps*	504	1,499	-4.6	14.5	-2.2	3.0	DNA stationary phase protection protein, ferritin[114,115]	R[43,44]	Yes
STM14_998	STM0853	*bssR*	384	8,127	-5.3	32.4	-3.2	3.1	BssR/YliH biofilm formation regulator[116]	R[43]	

Gene identification and annotations are from KEGG (http://www.genome.jp/kegg/kegg2.html) and the indicated references.

[a] : RNA-seq data included for comparison are from Lévi-Meyreuis *et al.* [16]

[b] : Detailed MS data are provided in Supplementary Datasets S2 and S3. Fold changes (log_2) in expression levels between the Δ*rpoS* mutant and the wild-type strains are indicated with the p-values ($-log_{10}$). ND : protein Not Detected.

[c]: R: reduced; I: increased

Table S2. Bacterial strains and plasmids used in this study.

Strain or Plasmid	Characteristics	Source or reference
VF6910	ATCC14028 *Salmonella enterica* serovar Typhimurium, wild-type strain	American Type Culture Collection
VF7928	ATCC14028 Δ*rpoS*::Cm	[4]
VF8158	VF7928 with the Cm cassette eliminated	[4]
VFC331	ATCC14028 Δ*rpoS* (scarless in frame deletion of *rpoS*)	[16]
VF8461	MA7224	[70]gift of N. Figueroa-Bossi
VF7985	ATCC14028 Δ*prpB*::Cm	This study
VFC47	VF8158 Δ*prpB*::Cm	This study
VFA693	MA7791 Δ*hfq67*::*cat*	[117]gift of N. Figueroa-Bossi
VFA714	ATCC14028 Δ*hfq67*::*cat*	This study
VFC45	ATCC14028 STM14_0421-3xflag::Km	This study
VFD182	ATCC14028 STM14_0460-3xflag::Km	This study
VFB990	ATCC14028 STM14_1271-3xflag::Km	This study
VFB572	ATCC14028 STM14_1275-3xflag::Km	This study
VFB818	ATCC14028 STM14_1829-3xflag::Km	This study
VFB503	ATCC14028 STM14_2091-3xflag::Km	This study
VFF503	ATCC14028 STM14_2173-3xflag::Km	This study
VFD106	ATCC14028 STM14_2189-3xflag::Km	This study
VFD100	ATCC14028 STM14_2239-3xflag::Km	This study
VFD108	ATCC14028 STM14_2405-3xflag::Km	This study
VFD190	ATCC14028 STM14_2409-3xflag::Km	This study
VFD926	ATCC14028 STM14_3597-3xflag::Km	This study
VFD112	ATCC14028 *yohP*-3xflag::Km	This study
VFD94	ATCC14028 STM14_4398-3xflag::Km	This study
VFD192	ATCC14028 STM14_4446-3xflag::Km	This study
VFB987	ATCC14028 STM14_5097-3xflag::Km	This study
VFD194	ATCC14028 STM14_5292-3xflag::Km	This study
VFB927	ATCC14028 STM14_5481-3xflag::Km	This study
VFF628	ATCC14028 *eutE*-3xflag::Km	This study
VFF622	ATCC14028 *pduO*-3xflag::Km	This study
VFF626	ATCC14028 *pduQ*-3xflag::Km	This study
VFF616	ATCC14028 *fucU*-3xflag::Km	This study
VFF618	ATCC14028 *fucA*-3xflag::Km	This study
VFF620	ATCC14028 *fucO*-3xflag::Km	This study
VFF638	ATCC14028 *mgsA*-3xflag::Km	This study
VFF642	ATCC14028 STM14_5317-3xflag::Km	This study
VFF640	ATCC14028 STM14_5326-3xflag::Km	This study
VFD71	VFC331 STM14_0421-3xflag::Km	This study
VFD183	VFC331 STM14_0460-3xflag::Km	This study
VFD68	VFC331 STM14_1271-3xflag::Km	This study
VFD64	VFC331 STM14_1275-3xflag::Km	This study
VFD65	VFC331 STM14_1829-3xflag::Km	This study
VFD63	VFC331 STM14_2091-3xflag::Km	This study
VFF504	VFC331 STM14_2173-3xflag::Km	This study
VFD107	VFC331 STM14_2189-3xflag::Km	This study
VFD101	VFC331 STM14_2239-3xflag::Km	This study
VFD109	VFC331 STM14_2405-3xflag::Km	This study
VFD191	VFC331 STM14_2409-3xflag::Km	This study
VFD66	VFC331 STM14_3597-3xflag::Km	This study
VFD113	VFC331 *yohP*-3xflag::Km	This study
VFD95	VFC331 STM14_4398-3xflag::Km	This study
VFD193	VFC331 STM14_4446-3xflag::Km	This study
VFD69	VFC331 STM14_5097-3xflag::Km	This study
VFD195	VFC331 STM14_5292-3xflag::Km	This study
VFD67	VFC331 STM14_5481-3xflag::Km	This study
VFF629	VFC331 *eutE*-3xflag::Km	This study
VFF623	VFC331 *pduO*-3xflag::Km	This study
VFF627	VFC331 *pduQ*-3xflag::Km	This study

VFF617	VFC331 *fucU*-3xflag::Km	This study
VFF619	VFC331 *fucA*-3xflag::Km	This study
VFF621	VFC331 *fucO*-3xflag::Km	This study
VFF639	VFC331 *mgsA*-3xflag::Km	This study
VFF643	VFC331 STM14_5317-3xflag::Km	This study
VFF641	VFC331 STM14_5326-3xflag::Km	This study
VFD320	ATCC14028 *prpE-lac*36	This study
VFD328	ATCC14028 *prpE-lac*40	This study
VFF544	ATCC14028 STM14_5292-*lac*36	This study
VFF435	ATCC14028 STM14_5292-*lac*40	This study
VFF706	ATCC14028 STM14_1932-*lac*40	This study
VFF702	ATCC14028 STM14_3199-*lac*40	This study
VFF710	ATCC14028 STM14_5469-*lac*40	This study
VFF698	ATCC14028 STM14_5479-*lac*40	This study
VFD321	VF8158 *prpE-lac*36	This study
VFD329	VF8158 *prpE-lac*40	This study
VFF545	VF8158 STM14_5292-*lac*36	This study
VFF436	VF8158 STM14_5292-*lac*40	This study
VFF707	VF8158 STM14_1932-*lac*40	This study
VFF703	VF8158 STM14_3199-*lac*40	This study
VFF711	VF8158 STM14_5469-*lac*40	This study
VFD321	VF8158 *prpE-lac*36	This study
VFD329	VF8158 *prpE-lac*40	This study
VFF545	VF8158 STM14_5292-*lac*36	This study
VFF436	VF8158 STM14_5292-*lac*40	This study
VFF707	VF8158 STM14_1932-*lac*40	This study
VFF703	VF8158 STM14_3199-*lac*40	This study
VFF711	VF8158 STM14_5469-*lac*40	This study
VFF699	VF8158 STM14_5479-*lac40*	This study
VFF583	ATCC14028 *prpE*-3xflag::Km	This study
VFF584	VFC331 *prpE*-3xflag::Km	This study
VFF972	VFA714 *prpE*-3xflag::Km	This study
VFF978	VFA714 *eutE*-3xflag::Km	This study
VFF976	VFA714 *pduO*-3xflag::Km	This study
VFF977	VFA714 *pduQ*-3xflag::Km	This study
VFF974	VFA714 *fucA*-3xflag::Km	This study
VFF979	VFA714 *mgsA*-3xflag::Km	This study
VFF981	VFA714 STM14_5317-3xflag::Km	This study
VFF751	ATCC14028 *pduO*-3xflag-*lac36*	This study
VFF755	ATCC14028 *pduQ*-3xflag-*lac36*	This study
VFF753	ATCC14028 *pduN*-3xflag-*lac36*	This study
VFF767	ATCC14028 *mgsA*-3xflag-*lac3*	This study
VFF747	ATCC14028 *fucA*-3xflag-*lac36*	This study
VFF745	ATCC14028 *fucU*-3xflag-*lac36*	This study
VFF771	ATCC14028 STM14_5317-3xflag-*lac36*	This study
VFF752	VF8158 *pduO*-3xflag-*lac36*	This study
VFF756	VF8158 *pduQ*-3xflag-*lac36*	This study
VFF754	VF8158 *pduN*-3xflag-*lac36*	This study
VFF768	VF8158 *mgsA*-3xflag-*lac3*	This study
VFF748	VF8158 *fucA*-3xflag-*lac36*	This study
VFF746	VF8158 *fucU*-3xflag-*lac36*	This study
FF772	VF8158 STM14_5317-3xflag-*lac36*	This study
**Plasmids**		
pACYC184	Cloning vector, Cm^R, Tet^R	[118]
pSTK4	*rpoS* cloned into pACYC184, Cm^R	[119]

Table S3. Oligonucleotides used in this study.

Name	Sequence (5' – 3')	Construction
STM14_0421-3FlagFw	CAGAGTCGGGCGATAAGCCTGACAGCCAGCCGCAAAAAA AATCGGACTACAAAGACCATGACGG	STM14_0421-3xFlag::Km
STM14_0421-3FlagRv	CGAAACGGTTATTTTCCCCAAACGTATAAACGTCGGGGAA TATCCACATATGAATATCCTCCTTAG	STM14_0421-3xFlag::Km
STM14_0460-3FlagFw	AGCGATGGATGCGAAAAATCGTTACGAAGATCCGGATAAA TCAGACTACAAAGACCATGACGG	STM14_0460-3xFlag::Km
STM14_0460-3FlagRv	TTTAGGGGTACTCAGAATCCCTTCCGAGTGGTTCATGCGTC TACATATGAATATCCTCCTTAG	STM14_0460-3xFlag::Km
STM14_1271-3FlagFw	GACTCGCTCTTCTGGGGCGAACAAATCATCGAACGTAAAA ACGTAGACTACAAAGACCATGACGG	STM14_1271-3xFlag::Km
STM14_1271-3FlagRv	GAACACTGCTGCAAAATAGCCATATGCCCCCGCGCAGGCG GGGGCGCATATGAATATCCTCCTTAG	STM14_1271-3xFlag::Km
STM14_1275-3FlagFw	CGAAGCAGGCAAAAAAGGGGGCAAAAGCAGTAACCGTAA TAGCGACTACAAAGACCATGACGG	STM14_1275-3xFlag::Km
STM14_1275-3FlagRv	GACACTTTTTAGCGCGAAACGCTCAGCGTCTTGCTGTTGTG ACATATGAATATCCTCCTTAG	STM14_1275-3xFlag::Km
STM14_1829-3FlagFw	GGTGGCCAAAATAGCCACAGTGGTGGACGGAAATCCGGCA ATGACTACAAAGACCATGACGG	STM14_1829-3xFlag::Km
STM14_1829-3FlagRv	CGTATGTGCAATATTTCAGAATATATTTATGAAATATCAGC ACATATGAATATCCTCCTTAG	STM14_1829-3xFlag::Km
STM14_2091-3FlagFw	GGTGGTCAGAATAGTCACGGCGGACGTAAATCCGATAATT CCGACTACAAAGACCATGACGG	STM14_2091-3xFlag::Km
STM14_2091-3FlagRv	CTCGCAGATTGCTTGATAAAAGCATGTGTTATATTTACATT ACCATATGAATATCCTCCTTAG	STM14_2091-3xFlag::Km
STM14_2173-3FlagFw	CGCGATCGTGGTGCTGGTGATTTTCCGCCTTCTGCGACGCG GCGACATCAAAGACCATGACGG	STM14_2173-3xFlag::Km
STM14_2173-3FlagRv	CCGGTGCTGACCGGGTCAACGCTTACACGTTCTGCAGCGG TTACATATGAATATCCTCCTTAG	STM14_2173-3xFlag::Km
STM14_2189-3FlagFw	GACACCCGAAGACGAAGGAAAAAATCCAAAGAAAAACCA AAAGGACTACAAAGACCATGACGG	STM14_2189-3xFlag::Km
STM14_2189-3FlagRv	GCCTCCAACGAACCATAGTTATTAAAGATACCCTACAGCA CTACATATGAATATCCTCCTTAG	STM14_2189-3xFlag::Km
STM14_2239-3FlagFw	TCATTACGATCCTAAATCTGATGCCTGGGTCATGCGTCTTG CCGACTACAAAGACCATGACGG	STM14_2239-3xFlag::Km
STM14_2239-3FlagRv	GAAGAATAAATAACCCGCCTGGCGACGGGTTCTTTTTGAG TCACATATGAATATCCTCCTTAG	STM14_2239-3xFlag::Km
STM14_2405-3FlagFw	CGAGCTGGATATTCATGATTTTAGTGTAACAGAAGTAAAC CGTGACTACAAAGACCATGACGG	STM14_2405-3xFlag::Km
STM14_2405-3FlagRv	GTATCCCGACCCGTAGGGCCGGGATTTTTTTCGGCCATTTT TACATATGAATATCCTCCTTAG	STM14_2405-3xFlag::Km
STM14_2409-3FlagFw	GCTCGTGCCAGGCAAGGAAAGGCGCGTGCGGGACGAAGC CCGAGACTACAAAGACCATGACGG	STM14_2409-3xFlag::Km
STM14_2409-3FlagRv	TTCTCCACAGCAAAACGCCCGGCATTAACCGGGCGTTTTGT CACATATGAATATCCTCCTTAG	STM14_2409-3xFlag::Km
STM14_3597-3FlagFw	GCAGATCAATCGTACTGACGTGAAAGAGATGGTGGCTCTG GAAAACGACTACAAAGACCATGACGG	STM14_3597-3xFlag::Km
STM14_3597-3FlagRv	GTGATTAATGTAGCACCGCCATATTGCGGTGCTTTTTTTTG TATAACCATATGAATATCCTCCTTAG	STM14_3597-3xFlag::Km
STnc1330-FlagFw	GATTGGGCTGTTGGTGGTAACGGGCGTGTTTAAGATGATTT TCGACTACAAAGACCATGACGG	*yohP*-3xFlag::Km
STnc1330-3FlagRv	ACGTGTGCCGGGCAGATACTATCGCTGCCCGGCGCAGGAA TCACATATGAATATCCTCCTTAG	*yohP*-3xFlag::Km
STM14_4398-3FlagFw	CCTGATACAGGCGAACCCACGCTTAAGTAAGCAATTGATG GAGGACTACAAAGACCATGACGG	STM14_4398-3xFlag::Km
STM14_4398-3FlagRv	GTCCTTACGCAGGACCGTTAAAAACAGAAAGGGGTAAAAA TTACATATGAATATCCTCCTTAG	STM14_4398-3xFlag::Km
STM14_4446-3FlagFw	ATATTACCGGCGGCTGTACCGACCAAAAGAAGAAGAAAG AGGTGACTACAAAGACCATGACGG	STM14_4446-3xFlag::Km
STM14_4446-3FlagRv	AATCAGCTTAAAAAAGGCCTTTCAAAAGTAAATCCCGCTG TTACATATGAATATCCTCCTTAG	STM14_4446-3xFlag::Km
STM14_5097-3FlagFw	GGAAAAAGAGGTTGTTGACTGGGAAACCCGTAACAACTAT CGCTGGGACTACAAAGACCATGACGG	STM14_5097-3xFlag::Km
STM14_5097-3FlagRv	CATAAGCAAACAGGGGGAAGCACATCCATGTACTCCTGGT AGGGGAACATATGAATATCCTCCTTAG	STM14_5097-3xFlag::Km
STM14_5292-3FlagFw	TGAAGTTAACCGTCAGGTTATGCGTCTGCAAACTGAGATG GCGGACTACAAAGACCATGACGG	STM14_5292-3xFlag::Km
STM14_5292-3FlagRv	TTTTTTTTTACTTTTTTAACCTACTGCATAGCACTTTTGGTT ACATATGAATATCCTCCTTAG	STM14_5292-3xFlag::Km
STM14_5481-3FlagFw	CGTGCTCTTCCTGGTCAGCCTGTTCATGGGCCGTAAACGAC CCGACTACAAAGACCATGACGG	STM14-5481-3xFlag::Km

STM14_5481-3FlagRv	GTAAAAGCCAGTCCGCTGGACTGGCTTGATAGCAATATAT CTTTACAGACATATGAATATCCTCCTTAG	STM14-5481-3xFlag::Km
prpE-P1-2	GGAGAGCGTGATGTCTTTTAGCGAATTTTATCAGCGTTCCA TTGTGTAGGCTGGAGCTGCTTC	transcriptional *prpE-lac*36 fusion
prpE-P2-2	CAGTCGCTGAGTCTAACCCGTTGCCGAACGCGGCTTATCCG GCCATATGAATATCCTCCTTAG	transcriptional *prpE-lac*36 fusion
prpE-P1R-1	CAGTCGCTGAGTCTAACCCGTTGCCGAACGCGGCTTATCCG GCGTGTAGGCTGGAGCTGCTTC	translational *prpE-lac40* fusion
prpE-P4-1	GGAGAGCGTGATGTCTTTTAGCGAATTTTATCAGCGTTCCA TTATTCCGGGGATCCGTCGACC	translational *prpE-lac40* fusion
prpB-P1	CGCCGGGGCAGGCATTTCGCGCCGCGCTCGCTAAAGAGAA TCCGTGTAGGCTGGAGCTGCTTC	Δ*prpB*::Cm
prpB-P2	CCTGTACAGCGCGTCCAGCTTCTCTTCGAACTGGTAGTAAT TGCATATGAATATCCTCCTTAG	Δ*prpB*::Cm
eutE-3FlagFw	GCGGCTGCGTCGATGCGTGCTGGTGGATGCGTTTCGCATTG TAGACTACAAAGACCATGACGG	*eutE*-3xFlag::Km
eutE-3FlagRv	GGTGAGCCAGAGTTGTTCGTCGTGCGCCATGAGTCATCCCT TACATATGAATATCCTCCTTAG	*eutE*-3xFlag::Km
pduO-3FlagFw	AGCACAGACCGCCATCGCGGCTATTAACGTGGGAACTCAT CAAGACTACAAAGACCATGACGG	*pduO*-3xFlag::Km
pduO-3FlagRv	CTAAGAATGGTGCGAATCAGGGTTTCGAGTTCAGAAGTAT TCACATATGAATATCCTCCTTAG	*pduO*-3xFlag::Km
pduQ-3FlagFw	GGCCAATGCCGAAGCCATTCGGGAACTGCTGGAGGAACTG CTAGACTACAAAGACCATGACGG	*pduQ*-3xFlag::Km
pduQ-3FlagRv	TCGGCGCTAAGGGACATTTCAACGCTGTTGATGGCGGTGC TCACATATGAATATCCTCCTTAG	*pduQ*-3xFlag::Km
fucU-3FlagFw	CGCGAAGTACGGAAATATTCTTTTAAAAAAAGGGGTAACG CCGGACTACAAAGACCATGACGG	*fucU*-3xFlag::Km
fucU-3FlagRv	TCGATGAACGCCCGCGTCATGCGGGCGCACCGGCAAGAGA TTACATATGAATATCCTCCTTAG	*fucU*-3xFlag::Km
fucA-3FlagFw	CGTACTGGAGAAATTTAAAACTTACGGATTACGTATTGAA GAGGACTACAAAGACCATGACGG	*fucA*-3xFlag::Km
fucA-3FlagRv	ATTCAGAATCATTCTGTTCGCCATCGCCTGTCTCCTGACAT CACATATGAATATCCTCCTTAG	*fucA*-3xFlag::Km
fucO-3FlagFw	AGCGAGTCTGGCGGACATTGTCGAACTGTATCATACCGCC TGGGACTACAAAGACCATGACGG	*fucO*-3xFlag::Km
fucO-3FlagRv	CGAAATGTAGAGCGGATAAGCGCAGCGCCATCCGGCAAA ATTACATATGAATATCCTCCTTAG	*fucO*-3xFlag::Km
mgsA-3FlagFw	TCTTATTCCGGATTATGCGCGTTATCTGGCCGAGCGCCTGA AAGACTACAAAGACCATGACGG	*mgsA*-3xFlag::Km
mgsA-3FlagRv	CCTAAAGCGCGGGCGGTACAGCATCCCGCCCGCGTAGCGT TTACATATGAATATCCTCCTTAG	*mgsA*-3xFlag::Km
prpE-3FlagFw	CGATCCCGCGTCGTTGCAGCAAATTCGCCAGGCGATCGAA GAAGACTACAAAGACCATGACGG	*prpE*-3xFlag::Km
prpE-3FlagRv	AGTCTAACCCGTTGCCGAACGCGGCTTATCCGGCTTACGGC TACATATGAATATCCTCCTTAG	*prpE*-3xFlag::Km
STM14_5317-3FlagFw	TACCGAGATTGTTGAATTACCCTCAAAACCTGATTTCTACA AAGACTACAAAGACCATGACGG	STM14_5317-3xFlag::Km
STM14_5317-3FlagRv	AAGCATCGCTATCCGGCAGGAGTTTTAATACGCGGAAGGT TTACATATGAATATCCTCCTTAG	STM14_5317-3xFlag::Km
STM14_5326-3FlagFw	AATAGAGGAACAAATTAATCGCAGCGTGTCATTGCTGCTG CAAGACTACAAAGACCATGACGG	STM14_5326-3xFlag::Km
STM14_5326-3FlagRv	GCTGCGGAAACTGAAAACGGTCACCTGGAAATTAGCTGTA TCACATATGAATATCCTCCTTAG	STM14_5326-3xFlag::Km
1932fin-P1R	CCCGCCAACATGCGCAGCAAGTACTCAAAGGTTGAACACC TCAGTGTAGGCTGGAGCTGCTTC	translational STM14_1932-*lac*40 fusion
1932fin-P4	AGGACTTTTGGGTCTGATCTCGTTGAGATTCGGCTGGTTTG CTATTCCGGGGATCCGTCGACC	translational STM14_1932-*lac*40 fusion
3199fin-P1R	AAAAAAGGCCGCCGAATGGCAGCCTCAAATGGAATATGTA TTAGTGTAGGCTGGAGCTGCTTC	translational STM14_3199-*lac*40 fusion
3199fin-P4	CGGAGAGGCTGAGATTCTGGCGGGACCGTTAGAACCTCCA ATTATTCCGGGGATCCGTCGACC	translational STM14_3199-*lac*40 fusion
5469fin-P1R	CTCGCACCAATTATCTTATCCTTCCTTTGTCTCTTCATTTTC AGTGTAGGCTGGAGCTGCTTC	translational STM14_5469-*lac*40 fusion
5469fin-P4	TATTGGCGGCATGATGTTGGCAATGATGAACTGGAATCAG GGAATTCCGGGGATCCGTCGACC	translational STM14_5469-*lac*40 fusion
5479fin-P1R	CTCAGGCAACACGAGGTTGCATTGCTGAATGCGGTGAAAC TCAGTGTAGGCTGGAGCTGCTTC	translational STM14_5479-*lac*40 fusion
5479fin-P4	CAAATTCACGAATGTGATGCCAGTCATTGACTTCAGAAAC CGGATTCCGGGGATCCGTCGACC	translational STM14_5479-*lac*40 fusion

Supplementary Datasets

Dataset S1. Putative homologs of small-uncharacterized σ^S-dependent genes.

Dataset S2. nLC-MS/MS data.

Dataset S3. The σ^S–dependent proteome of *S.* Typhimurium ATCC14028 and comparison with published transcriptomic data.

Supplementary References

67 Datsenko, K. A. & Wanner, B. L. One-step inactivation of chromosomal genes in *Escherichia coli* K-12 using PCR products. *Proc Natl Acad Sci USA* **97**, 6640-6645, doi:10.1073/pnas.120163297 (2000).

68 Gerlach, R. G., Jackel, D., Holzer, S. U. & Hensel, M. Rapid oligonucleotide-based recombineering of the chromosome of *Salmonella enterica*. *Appl Environ Microbiol* **75**, 1575-1580, doi:10.1128/AEM.02509-08 (2009).

69 Ellermeier, C. D., Janakiraman, A. & Slauch, J. M. Construction of targeted single copy *lac* fusions using lambda Red and FLP-mediated site-specific recombination in bacteria. *Gene* **290**, 153-161 (2002).

70 Uzzau, S., Figueroa-Bossi, N., Rubino, S. & Bossi, L. Epitope tagging of chromosomal genes in *Salmonella*. *Proc Natl Acad Sci USA* **98**, 15264-15269, doi:10.1073/pnas.261348198 (2001).

71 Robbe-Saule, V., Lopes, M. D., Kolb, A. & Norel, F. Physiological effects of Crl in *Salmonella* are modulated by sigmaS level and promoter specificity. *J Bacteriol* **189**, 2976-2987, doi:10.1128/JB.01919-06 (2007).

72 Monteil, V. *et al.* Crl binds to domain 2 of sigma(S) and confers a competitive advantage on a natural *rpoS* mutant of *Salmonella enterica* serovar Typhi. *J Bacteriol* **192**, 6401-6410, doi:10.1128/JB.00801-10 (2010).

73 Altschul, S. F. *et al.* Gapped BLAST and PSI-BLAST: a new generation of protein database search programs. *Nucleic Acids Res* **25**, 3389-3402 (1997).

74 Pearson, W. R. Effective protein sequence comparison. *Methods Enzymol* **266**, 227-258 (1996).

75 Pearson, W. R. Rapid and sensitive sequence comparison with FASTP and FASTA. *Methods Enzymol* **183**, 63-98 (1990).

76 Katoh, K., Kuma, K., Toh, H. & Miyata, T. MAFFT version 5: improvement in accuracy of multiple sequence alignment. *Nucleic Acids Res* **33**, 511-518, doi:10.1093/nar/gki198 (2005).

77 Eddy, S. R. A new generation of homology search tools based on probabilistic inference. *Genome Inform* **23**, 205-211 (2009).

78 Iglewicz, B. & Hoaglin, D. How to detect and handle outliers. *ASQC Quality Press* (1993).

79 Monot, M., Orgeur, M., Camiade, E., Brehier, C. & Dupuy, B. COV2HTML: a visualization and analysis tool of bacterial next generation sequencing (NGS) data for postgenomics life scientists. *Omics* **18**, 184-195, doi:10.1089/omi.2013.0119 (2014).

80 Thompson, J. D., Higgins, D. G. & Gibson, T. J. CLUSTAL W: improving the sensitivity of progressive multiple sequence alignment through sequence weighting, position-specific gap penalties and weight matrix choice. *Nucleic Acids Res* **22**, 4673-4680 (1994).

81 Tunnacliffe, A. & Wise, M. J. The continuing conundrum of the LEA proteins. *Naturwissenschaften* **94**, 791-812, doi:10.1007/s00114-007-0254-y (2007).

82 Brock, M., Maerker, C., Schutz, A., Volker, U. & Buckel, W. Oxidation of propionate to pyruvate in *Escherichia coli.* Involvement of methylcitrate dehydratase and aconitase. *European J Biochem* **269**, 6184-6194 (2002).

83 Simonte, F. M., Dotsch, A., Galego, L., Arraiano, C. & Gescher, J. Investigation on the anaerobic propionate degradation by *Escherichia coli* K12. *Mol Microbiol* **103**, 55-66, doi:10.1111/mmi.13541 (2017).

84 Eletsky, A. *et al.* Structural and functional characterization of DUF1471 domains of *Salmonella* proteins SrfN, YdgH/SssB, and YahO. *PloS One* **9**, e101787, doi:10.1371/journal.pone.0101787 (2014).
85 Ibanez-Ruiz, M., Robbe-Saule, V., Hermant, D., Labrude, S. & Norel, F. Identification of RpoS (sigma(S))-regulated genes in *Salmonella enterica* serovar typhimurium. *J Bacteriol* **182**, 5749-5756 (2000).
86 Guinote, I. B. *et al.* Breaking through the stress barrier: the role of BolA in Gram-negative survival. *World J Microbiol Biotechnol* **30**, 2559-2566, doi:10.1007/s11274-014-1702-4 (2014).
87 Patridge, E. V. & Ferry, J. G. WrbA from *Escherichia coli* and *Archaeoglobus fulgidus* is an NAD(P)H:quinone oxidoreductase. *J Bacteriol* **188**, 3498-3506, doi:10.1128/JB.188.10.3498-3506.2006 (2006).
88 Figueira, R. *et al.* Adaptation to sustained nitrogen starvation by *Escherichia coli* requires the eukaryote-like serine/threonine kinase YeaG. *Sci Rep* **5**, 17524, doi:10.1038/srep17524 (2015).
89 Kim, B. H. *et al.* The formation of cyclopropane fatty acids in *Salmonella enterica* serovar Typhimurium. *Microbiology* **151**, 209-218, doi:10.1099/mic.0.27265-0 (2005).
90 Frohlich, K. S., Papenfort, K., Fekete, A. & Vogel, J. A small RNA activates CFA synthase by isoform-specific mRNA stabilization. *EMBO J* **32**, 2963-2979, doi:10.1038/emboj.2013.222 (2013).
91 Osman, D. *et al.* The copper supply pathway to a *Salmonella* Cu,Zn-superoxide dismutase (SodCII) involves P(1B)-type ATPase copper efflux and periplasmic CueP. *Mol Microbiol* **87**, 466-477, doi:10.1111/mmi.12107 (2013).
92 Pilonieta, M. C., Erickson, K. D., Ernst, R. K. & Detweiler, C. S. A protein important for antimicrobial peptide resistance, YdeI/OmdA, is in the periplasm and interacts with OmpD/NmpC. *J Bacteriol* **191**, 7243-7252, doi:10.1128/JB.00688-09 (2009).
93 Lesniak, J., Barton, W. A. & Nikolov, D. B. Structural and functional features of the *Escherichia coli* hydroperoxide resistance protein OsmC. *Protein Sci* **12**, 2838-2843, doi:10.1110/ps.03375603 (2003).
94 Jarrett, J. T. & Lansbury, P. T., Jr. Amyloid fibril formation requires a chemically discriminating nucleation event: studies of an amyloidogenic sequence from the bacterial protein OsmB. *Biochemistry* **31**, 12345-12352 (1992).
95 Jung, J. U., Gutierrez, C., Martin, F., Ardourel, M. & Villarejo, M. Transcription of *osmB*, a gene encoding an *Escherichia coli* lipoprotein, is regulated by dual signals. Osmotic stress and stationary phase. *J Biol Chem* **265**, 10574-10581 (1990).
96 Ding, Q., Kusano, S., Villarejo, M. & Ishihama, A. Promoter selectivity control of *Escherichia coli* RNA polymerase by ionic strength: differential recognition of osmoregulated promoters by E sigma D and E sigma S holoenzymes. *Mol Microbiol* **16**, 649-656 (1995).
97 Osborne, M. J., Siddiqui, N., Iannuzzi, P. & Gehring, K. The solution structure of ChaB, a putative membrane ion antiporter regulator from *Escherichia coli*. *BMC Struct Biol* **4**, 9, doi:10.1186/1472-6807-4-9 (2004).
98 Kenyon, W. J. *et al.* Sigma(s)-Dependent carbon-starvation induction of pbpG (PBP 7) is required for the starvation-stress response in *Salmonella enterica* serovar Typhimurium. *Microbiology* **153**, 2148-2158, doi:10.1099/mic.0.2007/005199-0 (2007).
99 Yoshida, H. *et al.* YqjD is an inner membrane protein associated with stationary-phase ribosomes in *Escherichia coli*. *J Bacteriol* **194**, 4178-4183, doi:10.1128/JB.00396-12 (2012).

100 Kalliri, E., Mulrooney, S. B. & Hausinger, R. P. Identification of *Escherichia coli* YgaF as an L-2-hydroxyglutarate oxidase. *J Bacteriol* **190**, 3793-3798, doi:10.1128/JB.01977-07 (2008).

101 Metzner, M., Germer, J. & Hengge, R. Multiple stress signal integration in the regulation of the complex sigma S-dependent *csiD-ygaF-gabDTP* operon in *Escherichia coli*. *Mol Microbiol* **51**, 799-811 (2004).

102 Bernal-Cabas, M., Ayala, J. A. & Raivio, T. L. The Cpx envelope stress response modifies peptidoglycan cross-linking via the L,D-transpeptidase LdtD and the novel protein YgaU. *J Bacteriol* **197**, 603-614, doi:10.1128/JB.02449-14 (2015).

103 Ashraf, K. U. *et al.* The Potassium Binding Protein Kbp Is a Cytoplasmic Potassium Sensor. *Structure* **24**, 741-749, doi:10.1016/j.str.2016.03.017 (2016).

104 Navarre, C. & Goffeau, A. Membrane hyperpolarization and salt sensitivity induced by deletion of PMP3, a highly conserved small protein of yeast plasma membrane. *EMBO J* **19**, 2515-2524, doi:10.1093/emboj/19.11.2515 (2000).

105 De Block, J. *et al.* Yeast Pmp3p has an important role in plasma membrane organization. *J Cell Sci* **128**, 3646-3659, doi:10.1242/jcs.173211 (2015).

106 Raivio, T. L., Leblanc, S. K. & Price, N. L. The *Escherichia coli* Cpx envelope stress response regulates genes of diverse function that impact antibiotic resistance and membrane integrity. *J Bacteriol* **195**, 2755-2767, doi:10.1128/JB.00105-13 (2013).

107 Porcheron, G., Garenaux, A., Proulx, J., Sabri, M. & Dozois, C. M. Iron, copper, zinc, and manganese transport and regulation in pathogenic Enterobacteria: correlations between strains, site of infection and the relative importance of the different metal transport systems for virulence. *Front Cell Infect Microbiol* **3**, 90, doi:10.3389/fcimb.2013.00090 (2013).

108 Osman, D. & Cavet, J. S. Metal sensing in *Salmonella*: implications for pathogenesis. *Adv Microb Physiol* **58**, 175-232, doi:10.1016/B978-0-12-381043-4.00005-2 (2011).

109 Abdallah, J., Caldas, T., Kthiri, F., Kern, R. & Richarme, G. YhbO protects cells against multiple stresses. *J Bacteriol* **189**, 9140-9144, doi:10.1128/JB.01208-07 (2007).

110 Abdallah, J., Mihoub, M., Gautier, V. & Richarme, G. The DJ-1 superfamily members YhbO and YajL from *Escherichia coli* repair proteins from glycation by methylglyoxal and glyoxal. *Biochem Biophys Res Commun* **470**, 282-286, doi:10.1016/j.bbrc.2016.01.068 (2016).

111 Lee, C., Lee, J., Lee, J. Y. & Park, C. Characterization of the *Escherichia coli* YajL, YhbO and ElbB glyoxalases. *FEMS Microbiol lett* **363**, doi:10.1093/femsle/fnv239 (2016).

112 Bishop, R. E., Leskiw, B. K., Hodges, R. S., Kay, C. M. & Weiner, J. H. The entericidin locus of *Escherichia coli* and its implications for programmed bacterial cell death. *J Mol Biol* **280**, 583-596, doi:10.1006/jmbi.1998.1894 (1998).

113 Zheng, X., Ji, Y., Weng, X. & Huang, X. RpoS-dependent expression of OsmY in *Salmonella enterica* serovar typhi: activation under stationary phase and SPI-2-inducing conditions. *Curr Microbiol* **70**, 877-882, doi:10.1007/s00284-015-0802-1 (2015).

114 De Martino, M., Ershov, D., van den Berg, P. J., Tans, S. J. & Meyer, A. S. Single-Cell Analysis of the Dps Response to Oxidative Stress. *J Bacteriol* **198**, 1662-1674, doi:10.1128/JB.00239-16 (2016).

115 Yoo, A. Y. *et al.* Requirement of Fur for the full induction of Dps expression in *Salmonella enterica* serovar typhimurium. *J Microbiol Biotechnol* **17**, 1452-1459 (2007).

116 Domka, J., Lee, J. & Wood, T. K. YliH (BssR) and YceP (BssS) regulate *Escherichia coli* K-12 biofilm formation by influencing cell signaling. *Appl Environ Microbiol* **72**, 2449-2459, doi:10.1128/AEM.72.4.2449-2459.2006 (2006).
117 Figueroa-Bossi, N., Lemire, S., Maloriol, D., Balbontin, R., Casadesus, J. & Bossi, L. Loss of Hfq activates the sigmaE-dependent envelope stress response in *Salmonella enterica*. *Mol Microbiol* **62,** 838-852 (2006).
118 Chang, A. C. & Cohen, S. N. Construction and characterization of amplifiable multicopy DNA cloning vehicles derived from the P15A cryptic miniplasmid. *J Bacteriol* **134**, 1141-1156 (1978).
119 Kowarz, L., Coynault, C., Robbe-Saule, V. & Norel, F. The *Salmonella typhimurium katF* (*rpoS*) gene: cloning, nucleotide sequence, and regulation of *spvR* and *spvABCD* virulence plasmid genes. *J Bacteriol* **176**, 6852-6860 (1994).
